# Supplementary material for: Protocol for validation of the Global Scales for Early Development (GSED) for children under 3 years of age in seven countries
Source: BMJ Open. 2023 Jan 24;13(1):e062562. doi: 10.1136/bmjopen-2022-062562 (PMC9884878; doi:10.1136/bmjopen-2022-062562)
Supplement: Supplementary data [file bmjopen-2022-062562supp001.pdf]

Supplementary file 1 – Sample size calculations and sampling frame

The sample size calculation for reliability is based on a confidence interval (CI) approach and the desired accuracy for the lower bound of the CI for the ICC estimates. In an Analysis of Variance (ANOVA) with 2-way random effects on a single score with 2 observations per subject (following Shrout and Fleiss, 1979) (27) and with a two-sided 95% confidence interval and an expected ICC of 0.875, the lower confidence interval for the inter-rater reliability sample (N=90) = 0.852. With the same parameters but an expected ICC of 0.90 the lower confidence interval for the test-retest reliability sample (N=50) is 0.830. We expect the ICC to be higher for the test-retest reliability than the inter-rater reliability as inter-rater estimates contain all the sources of error in the test-retest estimates, plus additional error between assessors (14).

To assess concurrent validity, a sample size of 150 per site produces a two-sided 95% CI 0.15-0.44, when the estimate of Pearson's product-moment correlation is 0.30, with an equal spread of participants tested across age and sex. The CI will be narrower when the data are combined across all seven countries. To assess predictive validity a sample size of 404 produces a two-sided 95% CI 0.65-0.75 when the estimate of Pearson's product-moment correlation is 0.70 between individual scores at baseline and at the 6-month follow-up. Allowing 20% dropout at follow up, a sample size of approximately 500 participants is required.

Table S1. Sampling Frame

Sample size per site by age and sex for total population (n=1248) which includes a minimum subsample of healthy 'reference' children (n=522)

Age (Days)	Sex	Total Sample size	Minimum sub-sample of reference children	Predictive validity sample (6-month follow-up; age at baseline)	Reliability: Inter-rater	Reliability: Test-Retest	Concurrent validity
15-30	Male	40	20	8	2	1	4
	Female	40	20	8	2	1	2
31-61	Male	40	12	8	1	1	2
	Female	40	12	8	2	1	2
62-91	Male	40	10	8	2	1	2
	Female	40	10	8	1	0	4
92-122	Male	36	9	8	2	1	2
	Female	36	9	8	2	1	2
123-152	Male	32	8	8	1	1	2
	Female	32	8	8	2	1	2

153-183	Male	28	8	8	1	0	4
	Female	28	8	8	1	1	2
184-213	Male	25	7	8	2	1	2
	Female	25	7	8	1	0	2
214-244	Male	23	7	8	1	1	2
	Female	23	7	8	2	1	4
245-274	Male	21	6	8	1	1	2
	Female	21	6	8	1	1	2
275-304	Male	19	6	8	2	0	2
	Female	19	6	8	1	1	2
305-335	Male	17	6	8	1	1	4
	Female	17	6	8	2	0	2
336-365	Male	16	6	7	1	1	2
	Female	16	6	7	1	1	2
366-396	Male	14	6	7	2	1	2
	Female	14	6	7	1	1	4
397-426	Male	13	6	7	1	0	2
	Female	13	6	7	2	1	2
427-457	Male	12	5	7	1	1	2
	Female	12	5	7	1	0	2
458-487	Male	11	5	7	2	1	4
	Female	11	5	7	1	1	2
488-517	Male	11	5	7	1	1	2
	Female	11	5	7	2	1	2
518-548	Male	10	5	7	1	0	2
	Female	10	5	7	1	1	4
549-578	Male	9	5	7	2	1	2
	Female	9	5	7	1	0	2
579-609	Male	9	5	7	1	1	2
	Female	9	5	7	2	1	2
610-639	Male	9	5	7	1	1	4
	Female	9	5	7	1	1	2
640-670	Male	9	5	7	2	0	2
	Female	9	5	7	1	1	2
671-700	Male	9	5	7	1	1	2
	Female	9	5	7	2	0	4
701-730	Male	9	5	7	1	1	2
	Female	9	5	7	1	1	2
731-761	Male	9	5	7	2	1	2
	Female	9	5	7	1	1	2
762-791	Male	9	5	6	1	0	4
	Female	9	5	6	2	1	2
792-822	Male	9	5	6	1	1	2
	Female	9	5	6	1	0	2
823-852	Male	9	5	6	2	1	2
	Female	9	5	6	1	1	2

853-883	Male	9	5	6	1	1	2
	Female	9	5	6	2	1	2
884-913	Male	9	5	6	1	0	2
	Female	9	5	6	1	1	2
914-944	Male	9	5	6	2	1	2
	Female	9	5	6	1	0	2
945-974	Male	9	5	6	1	1	2
	Female	9	5	6	2	1	2
975-1004	Male	9	5	6	1	1	2
	Female	9	5	6	1	1	2
1005-1035	Male	9	5	6	2	0	2
	Female	9	5	6	1	1	2
1036-1065	Male	9	5	6	1	1	2
	Female	9	5	6	2	0	2
1066-1096	Male	9	5	6	1	1	2
	Female	9	5	6	1	1	2
1097-1126	Male	9	5	0	0	0	0
	Female	9	5	0	0	0	0
1127-1157	Male	9	5	0	0	0	0
	Female	9	5	0	0	0	0
1158-1187	Male	9	5	0	0	0	0
	Female	9	5	0	0	0	0
1188-1218	Male	9	6	0	0	0	0
	Female	9	6	0	0	0	0
1219-1248	Male	9	6	0	0	0	0
	Female	9	6	0	0	0	0
1249-1279	Male	9	7	0	0	0	0
	Female	9	7	0	0	0	0
**TOTAL**		1248	522	504	*99	**55	***166

*90 + ~10% Loss to follow up = 99; **50 + ~10% Loss to follow up = 55; ***150 + ~10% Loss to follow up = 166
