# Supplementary material for: Protocol for validation of the Global Scales for Early Development (GSED) for children under 3 years of age in seven countries
Source: BMJ Open. 2023 Jan 24;13(1):e062562. doi: 10.1136/bmjopen-2022-062562 (PMC9884878; doi:10.1136/bmjopen-2022-062562)
Supplement: Supplementary data [file bmjopen-2022-062562supp002.pdf]

## Supplementary file 2 – Visit schedule

### Table S2a.Visit Schedule for the GSED Validation Study (all sites except the Netherlands)

| **Main Study Only [No Sub-sample]** | **Inter- Rater Reliability Sub- Sample** | **Test- Retest Reliability Sub- Sample** | **Concurrent Sub- Sample 1 [LF First]** | **Concurrent Sub- Sample 2 [BSID III First]** |
|---|---|---|---|---|
| **Visit 1 [At Home]** | | | | |
| Eligibility and Consent | Eligibility and Consent | Eligibility and Consent | Eligibility and Consent | Eligibility and Consent |
| COVID Questionnaire | COVID Questionnaire | COVID Questionnaire | COVID Questionnaire | COVID Questionnaire |
| Contextual | Contextual | Contextual | Contextual | Contextual |
| GSED Short form [SF] | GSED Short form [SF] | GSED Short form [SF] | GSED Short form [SF] | GSED Short form [SF] |
| GSED Psychosocial form [PF] | GSED Psychosocial form [PF] | GSED Psychosocial form [PF] | GSED Psychosocial form [PF] | GSED Psychosocial form [PF] |
| HOME Inventory or Family Care Indicators (FCI) | HOME Inventory or Family Care Indicators (FCI) | HOME Inventory or Family Care Indicators (FCI) | HOME Inventory or Family Care Indicators (FCI) | HOME Inventory or Family Care Indicators (FCI) |
| Anthropometrics* | Anthropometrics* | Anthropometrics* | Anthropometrics* | Anthropometrics* |
| **Visit 2 [At home, clinic, or other setting within 48 hours of visit 1]** ***Note: For Concurrent Sample, the Visit is at the Clinic setting*** | | | | |
| Abbreviated Eligibility [ Coversheet] | Abbreviated Eligibility [ Coversheet] | Abbreviated Eligibility [ Coversheet] | Abbreviated Eligibility [ Coversheet] | Abbreviated Eligibility [ Coversheet] |
| GSED Long form [LF] | GSED Long form [LF] | GSED Long form [LF] | **GSED Long form [LF]** | **BSID III** |
| CPAS | CPAS | CPAS | CPAS | ---------------------- |
| PHQ9 | PHQ9 | PHQ9 | PHQ9 | --------------------- |
| Family support & Resilience Scale | Family support & Resilience Scale | Family support & Resilience Scale | Family support & Resilience Scale | ---------------------- |
| **Visit 3 [Setting and timing vary by sub-sample]** | | | | |
| Visit 3 not required | Visit 3 [At home, clinic or other setting where the LF was completed- **within 24** hours of the LF] | Visit 3 [At home, clinic or other setting where the LF was completed- this should happen **7 to 10 days after** LF] | Visit 3 [Clinic setting within **24- 72 hours** of the LF- can be done at same time as Visit 2 – taking child fatigue into consideration] | Visit 3 [Clinic setting within **24- 72 hours** of the BSID III - can be done at same time as Visit 2 – taking child fatigue into consideration] |
| | Abbreviated Eligibility [ Coversheet] | Abbreviated Eligibility [ Coversheet] | Abbreviated Eligibility [ Coversheet] | Abbreviated Eligibility [ Coversheet] |
| | GSED Short form [SF] | GSED Short form [SF] | **BSID III** | **GSED Long form [LF]** |
| | GSED Psychosocial form [PF] | GSED Psychosocial form [PF] | ---------------------- | CPAS |
| | GSED Long form [LF] | GSED Long form [LF] | ---------------------- | PHQ9 |
| | ---------------------- | ---------------------- | ---------------------- | Family support & Resilience Scale |

* Anthropometrics may be done either at visit 1 or visit 2

**Table S2b: Visit Schedule for the GSED Validation Study (the Netherlands only)**

| **Main Study Only [No Sub-sample]** | **Inter- Rater Reliability Sub- Sample** | **Test- Retest Reliability Sub- Sample** | **Concurrent Sub- Sample 1 [LF First]** | **Concurrent Sub- Sample 2 [BSID III First]** |
|---|---|---|---|---|
| **Session 1 [Online]** | | | | |
| Eligibility and Consent | Eligibility and Consent | Eligibility and Consent | Eligibility and Consent | Eligibility and Consent |
| Contextual | Contextual | Contextual | Contextual | Contextual |
| GSED Short form [SF] | GSED Short form [SF] | GSED Short form [SF] | GSED Short form [SF] | GSED Short form [SF] |
| GSED Psychosocial scale [PS] | GSED Psychosocial scale [PS] | GSED Psychosocial scale [PS] | GSED Psychosocial scale [PS] | GSED Psychosocial scale [PS] |
| **Visit 1 [At clinic within 48 hours of session1]** | | | | |
| Abbreviated Eligibility [ Coversheet] | Abbreviated Eligibility [ Coversheet] | Abbreviated Eligibility [ Coversheet] | Abbreviated Eligibility [ Coversheet] | Abbreviated Eligibility [ Coversheet] |
| GSED Long form [LF] | GSED Long form [LF] | GSED Long form [LF] | **GSED Long form [LF]** | **BSID III** |
| Anthropometrics | Anthropometrics | Anthropometrics | Anthropometrics | Anthropometrics |
| **Session 2 [Online, Test-Retest of SF/PSY within 7 to 10 days of online session 1]** | | | | |
| Abbreviated Eligibility [ Coversheet] | Abbreviated Eligibility [ Coversheet] | Abbreviated Eligibility [ Coversheet] | Abbreviated Eligibility [ Coversheet] | Abbreviated Eligibility [ Coversheet] |
| COVID Questionnaire | COVID Questionnaire | COVID Questionnaire | COVID Questionnaire | COVID Questionnaire |
| ---------------------- | ---------------------- | GSED Short form [SF] | ---------------------- | ---------------------- |
| ---------------------- | ---------------------- | GSED Psychosocial scale [PS] | ---------------------- | ---------------------- |
| CPAS | CPAS | CPAS | CPAS | CPAS |
| PHQ9 | PHQ9 | PHQ9 | PHQ9 | PHQ9 |
| Family support & Resilience Scale | Family support & Resilience Scale | Family support & Resilience Scale | Family support & Resilience Scale | Family support & Resilience Scale |
| Family Care Indicators (FCI) | Family Care Indicators (FCI) | Family Care Indicators (FCI) | Family Care Indicators (FCI) | Family Care Indicators (FCI) |
| **Visit 2 [At clinic, timing varies by sub-sample]** | | | | |
| Visit 2 not required | Visit 2 [**within 24** hours of the LF] | Visit 2 [**7 to 10 days after** LF] | Visit 2 [within **24- 72 hours** of the LF- can be done at same time as Visit 1 – taking child fatigue into consideration] | Visit 2 [within **24- 72 hours** of the BSID III - can be done at same time as Visit 1 – taking child fatigue into consideration] |
| | Abbreviated Eligibility [ Coversheet] | Abbreviated Eligibility [ Coversheet] | Abbreviated Eligibility [ Coversheet] | Abbreviated Eligibility [ Coversheet] |
| | GSED Long form [LF] | GSED Long form [LF] | **BSID III** | **GSED Long form [LF]** |
