# Supplementary material for: Protocol for validation of the Global Scales for Early Development (GSED) for children under 3 years of age in seven countries
Source: BMJ Open. 2023 Jan 24;13(1):e062562. doi: 10.1136/bmjopen-2022-062562 (PMC9884878; doi:10.1136/bmjopen-2022-062562)
Supplement: Author's manuscript [file bmjopen-2022-062562.draft_revisions.pdf]

BMJ Open is committed to open peer review. As part of this commitment we make the peer review history of every article we publish publicly available.

When an article is published we post the peer reviewers' comments and the authors' responses online. We also post the versions of the paper that were used during peer review. These are the versions that the peer review comments apply to.

The versions of the paper that follow are the versions that were submitted during the peer review process. They are not the versions of record or the final published versions. They should not be cited or distributed as the published version of this manuscript.

BMJ Open is an open access journal and the full, final, typeset and author-corrected version of record of the manuscript is available on our site with no access controls, subscription charges or pay-per-view fees (http://bmjopen.bmj.com).

If you have any questions on BMJ Open's open peer review process please email info.bmjopen@bmj.com

BMJ Open

Protocol for Validation of the Global Scales for Early Development (GSED) for Children under 3 Years of Age in Seven Countries

Journal:	*BMJ Open*
Manuscript ID	bmjopen-2022-062562
Article Type:	Protocol
Date Submitted by the Author:	04-Mar-2022
Complete List of Authors:	Cavallera, Vanessa; World Health Organization, Department of Mental Health and Substance Use<br>Lancaster, Gillian; Keele University School of Medicine<br>Gladstone, Melissa; University of Liverpool Institute of Translational Medicine, Women and Children's Health<br>Black, Maureen; RTI International; University of Maryland School of Medicine<br>McCray, Gareth; Keele University School of Medicine<br>Nizar, Ambreen; The Aga Khan University, Department of Peadiatrics<br>Ahmed, Salahuddin; Projahnmo Research Foundation<br>Dutta, Arup; Center for Public Health Kinetics<br>Anago, Romuald ; Innovations for Poverty Action<br>Brentani, Alexandra; University of Sao Paulo, Pediatrics<br>Jiang, Fan; Shanghai Children's Medical Center Affiliated to Shanghai Jiaotong University School of Medicine<br>Schönbeck, Yvonne; Netherlands Organization for Applied Scientific Research, Department of Child Health<br>McCoy, Dana; Harvard Graduate School of Education<br>Kariger, Patricia; University of California Berkeley School of Public Health<br>Weber, Ann; University of Nevada Reno<br>Raikes, Abbie; University of Nebraska Medical Center College of Public Health<br>Waldman, Marcus; University of Nebraska Medical Center College of Public Health<br>van Buuren, Stef; TNO, Department of Statistics<br>Kaur, Raghbir; World Health Organization<br>Maillard, Pérez; World Health Organization<br>Nisar, Muhammad; The Aga Khan University, Pediatrics and Child Health<br>Khanam, Rasheda; Johns Hopkins University, International Center for Maternal and Newborn Health, Department of International Health, Johns Hopkins Bloomberg School of Public Health<br>Sazawal, Sunil ; Centre for Public Health Kinetics<br>Zongo, Arsène; Innovations for Poverty Action<br>Pacifico Mercadante, Mariana; University of Sao Paulo, Pediatrics<br>Zhang, Yunting ; Shanghai Children's Medical Center Affiliated to Shanghai Jiaotong University School of Medicine<br>Roy, Arunangshu ; Projahnmo Research Foundation<br>Hepworth, Katelyn; University of Nebraska-Lincoln College of Education and Human Sciences

Fink, Günther ; Schweizerisches Tropen- und Public Health-Institut, Household Economics and Health System Research Unit
Rubio-Codina, Marta; Inter-American Development Bank
Tofail, Fahmida; International Centre for Diarrhoeal Disease Research Bangladesh, Centre for Nutrition & Food Security
Eekhout, Iris; TNO, Child Health
Seiden, Jonathan; Harvard Graduate School of Education
Norton, Rebecca; World Health Organization
Baqui, Abdullah; Johns Hopkins University Bloomberg School of Public Health
Zhao, Jin; Shanghai Children's Medical Center Affiliated to Shanghai Jiaotong University School of Medicine
Holzinger, Andreas; Innovations for Poverty Action
Detmar, Symone; TNO
Kembou, Samuel; Innovations for Poverty Action
Begum, Farzana; The Aga Khan University, Department of Peadiatrics
Jehan, Fyezah; The Aga Khan University, Paediatrics and Child Health
Dua, Tarun; World Health Organization
Janus, Magdalena; McMaster University, Offord Centre for Child Studies

Keywords: MENTAL HEALTH, Paediatric neurology < NEUROLOGY, Developmental neurology & neurodisability < PAEDIATRICS, PUBLIC HEALTH, EPIDEMIOLOGY

SCHOLARONE™ Manuscripts

BMJ Open

Title: Protocol for Validation of the Global Scales for Early Development (GSED) for Children under 3 Years of Age in Seven Countries

Authors: Cavallera V, Lancaster G, Gladstone M, Black MM, McCray G, Nizar A, Ahmed S, Dutta A, Anago R, Brentani A, Jiang F, Schönbeck Y, McCoy D, Kariger P, Weber A, Raikes A, Waldman M, van Buuren S, Kaur R, Pérez Maillard M, Nisar I, Khanam R, Sazawal S, Zongo A, Pacifico Mercadante M, Zhang Y, Roy AD, Hepworth K, Fink G, Rubio Codina M, Tofail F, Eekhout I, Seiden J, Norton R, Baqui AH, Zhao J, Holzinger A, Detmar S, Kembou S N, Begum F, Jehan F, Dua T, Janus M.

Affiliations:

Corresponding author: Dr Vanessa Cavallera, MD, MPH, World Health Organization (WHO), Brain Health Unit in Department of Mental Health and Substance Use, Av. Appia 20, 1202 Geneva, Switzerland. cavallerav@who.int

Prof Gillian Lancaster, PhD, MSc, BSc (Hons), Clinical Trials Unit, School of Medicine, Keele University, UK

Prof Melissa Gladstone, MD, International Child Health and Neurodevelopmental Paediatrics, Department of Women and Children's Health, Institute of Translational Medicine, University of Liverpool, Alder Hey Children's NHS Foundation Trust, UK.

Prof Maureen Black, PhD, Department of Pediatrics, University of Maryland School of Medicine, Baltimore, MD, USA and RTI International, Research Park, NC, USA

Dr Gareth McCray, MA, MRes, PhD, Research Associate, the School of Medicine, Keele University, Keele, UK.

Ambreen Nizar, BDS, MSc, Department of Peadiatrics, Aga Khan University, Karachi Pakistan.

Salahuddin Ahmed, MBBS, Projahnmo Research Foundation, Dhaka, Bangladesh

Arup Dutta, MBA, Center for Public Health Kinetics, New Delhi, India

Romuald Kouadio E Anago, MA, BA, Innovations for Poverty Action (IPA), Abidjan, Côte d'Ivoire

Dr. Alexandra Valeria Maria Brentani, PhD, Dept. of Pediatrics, University of Sao Paulo Medical School, Brazil

Prof. Fan Jiang, MD, PhD, Department of Developmental and Behavioral Pediatrics, National Children's Medical Center, Shanghai Children's Medical Center, affiliated to School of Medicine Shanghai Jiao Tong University, China

Dr. Yvonne Schönbeck, PhD, Msc, Dept. Child Health, Netherlands Organization for Applied Scientific Research (TNO), Leiden, The Netherlands

Dr Dana McCoy, PhD, Harvard Graduate School of Education, Cambridge, MA, USA

Dr Patricia Kariger, PhD CEGA (Center for Effective Global Action), School of Public Health, University of California, Berkeley, California, USA.

Dr Ann Weber, PhD, MPH, School of Public Health, University of Nevada, Reno, NV, USA

Dr Hilary Abigail Raikes, MPH, PhD, University of Nebraska Medical Center, College of Public Health, Omaha, NE, USA

Dr. Marcus Waldman, PhD, University of Nebraska Medical Center, College of Public Health, Omaha, NE, USA

Prof Stef van Buuren, PhD, Department of Child Health, Netherlands Organization for Applied Scientific Research TNO, Leiden, The Netherlands

For peer review only

Raghbir Kaur, MPH, DMD, MS, Consultant World Health Organization (WHO), Brain Health Unit in Department of Mental Health and Substance Use, Geneva, Switzerland

Michelle Pérez Maillard, MSc, Consultant World Health Organization (WHO), Brain Health Unit in Department of Mental Health and Substance Use, Geneva, Switzerland

Prof. Muhammad Imran Nisar, MBBS, MSc, Department of Peadiatrics, Aga Khan University, Karachi Pakistan.

Dr. Rasheda Khanam, MBBS, MPH, PhD, International Center for Maternal and Newborn Health, Department of International Health, Johns Hopkins University Bloomberg School of Public Health, Baltimore, MD, USA

Dr. Sunil Sazawal, MBBS, MPH, PhD, Center for Public Health Kinetics New Delhi, India

Mr. Arsène Zongo, MA, BA, Innovations for Poverty Action (IPA), Abidjan, Côte d'Ivoire

Dr. Mariana Pacifico Mercadante, MD, Dept. of Pediatrics, University of Sao Paulo Medical School, Brazil

Dr. Yunting Zhang, PhD, Child Health Advocacy Institute, National Children's Medical Center, Shanghai Children's Medical Center, affiliated to School of Medicine Shanghai Jiao Tong University, China

Dr. Arunangshu D. Roy, MBBS, Projahnmo Research Foundation, Dhaka, Bangladesh

Katelyn Hepworth, MA, University of Nebraska-Lincoln, College of Education and Human Sciences, NE, USA

Prof Günther Fink, PhD, Swiss Tropical and Public Health Institute, Basel, Switzerland

For peer review only

Dr Marta Rubio-Codina, PhD, Inter-American Development Bank, Washington, D.C., USA

Dr. Fahmida Tofail, MBBS, PhD, International Centre for Diarrhoeal Disease Research Icddr,b, Dhaka, Bangladesh

Dr. Iris Eekhout, PhD, MSc, Department of Child Health, Netherlands Organization for Applied Scientific Research TNO, Leiden, The Netherlands

Jonathan Seiden, EdM, Harvard Graduate School of Education, Cambridge, MA, USA

Rebecca Norton, Consultant World Health Organization (WHO), Brain Health Unit in Department of Mental Health and Substance Use, Geneva, Switzerland

Dr. Abdullah H. Baqui, MBBS, DrPH, Johns Hopkins Bloomberg School of Public Health, Baltimore, Maryland, USA

Dr. Jin Zhao, MD, PhD, Department of Developmental and Behavioral Pediatrics, National Children's Medical Center, Shanghai Children's Medical Center, affiliated to School of Medicine Shanghai Jiao Tong University, China

Andreas Holzinger, MA, BA, Innovations for Poverty Action (IPA), Abidjan, Côte d'Ivoire

Dr. Symone Detmar, PhD, Msc, Department of Child Health, Netherlands Organization for Applied Scientific Research TNO, Leiden, The Netherlands

Dr, Samuel Nzale Kembou, PhD, MA, BA, Innovations for Poverty Action (IPA), Abidjan, Côte d'Ivoire

Farzana Begum, MSc, Department of Peadiatrics, Aga Khan University, Karachi Pakistan.

For peer review only

Prof. Fyezah Jehan, MBBS, FCPS, MSc, Department of Peadiatrics, Aga Khan University, Karachi Pakistan.

Dr Tarun Dua, MD, World Health Organization (WHO), Brain Health Unit in Department of Mental Health and Substance Use, Geneva, Switzerland

Prof Magdalena Janus, MBBS, FCPS, MSc, Offord Centre for Child Studies, McMaster University, Hamilton ON, Canada.

Disclaimers:

The author is a member of the World Health Organization. The author alone is responsible for the views expressed in this publication and they do not necessarily represent the decisions, policy or views of the World Health Organization. [Applies to Cavallera V, Dua T, Kaur R, Pérez Maillard M and Norton R]

The views here presented do not represent the Inter-American Development Bank, its board of directors, or the countries it represents. [Applies to Rubio Codina M]

Wordcount: 4000

Keywords: Early childhood development, global, measurement, index, population, evaluation, validation, protocol, programmes.

For peer review only

For peer review only - http://bmjopen.bmj.com/site/about/guidelines.xhtml

ABSTRACT

Introduction. Children's early development is affected by caregiving experiences, with life-long health and wellbeing implications. Governments and civil societies need population-based measures to monitor children's early development and ensure that children receive the care needed to thrive. To this end, the World Health Organization (WHO) developed the Global Scales for Early Development (GSED) to measure children's early development (ages 0-3 years). The GSED includes three measures: 1) short form (SF) for population-evaluation (caregiver-report), 2) complementary long form (LF) for programmatic-evaluation (direct assessment), and 3) psychosocial form (PF) for psychosocial development evaluation (caregiver-report). The primary aim of this protocol is to validate the GSED SF and LF. Secondary aims are to create preliminary reference scores for the GSED SF and LF, validate an adaptive testing algorithm, and assess the feasibility and preliminary validity of the GSED PF.

Methods and Analysis. We will conduct the validation in seven countries varying in geography, language, culture and income through a one-year prospective design, combining cross-sectional and longitudinal methods with 1248 children per site, stratified by age and sex. The GSED generates an innovative common metric (Development-score: D-score) using the Rasch model and a development-for-age z-score (DAZ). We will evaluate six psychometric properties of the GSED SF and LF: concurrent validity, predictive validity at six months, convergent and discriminant validity, and test-retest and inter-rater reliability. We will evaluate measurement invariance by comparing differential item functioning (DIF) and differential test functioning (DTF) across sites.

Ethics and dissemination. This study has received ethical approval from the WHO (protocol GSED validation 004583 20.04.2020) and approval in each site. Study results

For peer review only

will be disseminated through webinars and publications from WHO, international organisations, academic journals, and conference proceedings.

Registration details: Open Science Framework (OSF) https://osf.io/ on 19/11/2021 (DOI 10.17605/OSF.IO/KX5T7; identifier: osf-registrations-kx5t7-v1)

ARTICLE SUMMARY

Strengths and limitations of this study

- The study collects validation data (n = 8736 children) for the Global Scales for Early Development (GSED) in seven countries that vary in geographic, linguistic, cultural and sociodemographic characteristics. The sampled populations are chosen to be diverse and are not nationally representative.
- The methods for the validation of GSED are systematic across sites and follow rigorous standard operating procedures based on the best scientific evidence available.
- A tablet-based App is used for data collection to make the administration of the GSED measures user-friendly, to reduce recording and transcribing errors, and to facilitate adaptive testing.
- The GSED SF and LF aims to include items that are culturally neutral and fit the Rasch model, which assumes that child development milestones are age-ordinal, to create D-scores. Psychosocial items are included in a separate measure (GSED psychosocial form [PF]) and cultural-specific items can be supplemented by countries.
- The three secondary aims (preliminary reference scores, an adaptive testing algorithm, and the feasibility and validity of the GSED PF), are exploratory and will require further research.

For peer review only - http://bmjopen.bmj.com/site/about/guidelines.xhtml

INTRODUCTION

Prenatal and early postnatal experiences have significant impacts on early childhood development (ECD) and can influence the accrual of health, well-being, and productivity throughout the life-course (1). To promote current and sustainable peace and prosperity, the United Nations has focused the Sustainable Development Goals (SDG) on improving children's outcomes in the early years through multiple targets. The most explicit target for young children is SDG 4 (Education goal), which requires reporting on the "proportion of children under 5 years of age who are developmentally on track in health, learning and psychosocial well-being, by sex" (2).

There are few valid measures that can be used globally to assess child development for children under three years of age. Current measures of ECD range from proxy measures (e.g., prevalence of country-level stunting and poverty) to detailed measures of individual performance on developmental tasks (3). The Early Childhood Development Index 2030 (ECDI 2030) (4) does not include children below two years of age. A recent review has identified the creation and validation of population-based instruments for assessing very young children as a global priority (5).

The Global Scales for Early Development (GSED) build on advances made by analyses of existing global datasets (6), and new data collection (7) that demonstrated the cross cultural applicability of items that measure young children's development. Three research teams (8) joined efforts to develop the GSED in response to the pressing need for instruments and metrics to measure ECD at population and programmatic levels across diverse parts of the world.

For peer review only

The Global Scales for Early Development (GSED)

The GSED consist of three open-access measures developed by a WHO-led team[1] to provide a standardized methodology for measuring the development of children aged 0-3 years (0-36 months) across diverse cultures and contexts (9, 10). They are developed for three objectives: 1) for population-level evaluation based on caregiver-report, GSED Short Form (SF); 2) for programmatic evaluation in combination with SF, direct child assessment, GSED Long Form (LF); and 3) for measuring psychosocial behaviours, caregiver-reported GSED Psychosocial Form (PF). The development and piloting of the GSED SF, LF, and PF are described elsewhere (9).

The GSED SF and LF produce metrics on the same age-ordinal scale and quantify the same latent construct. The Developmental Score (D-score) (see Box 1) underlies both measures and reflects children's overall development across multiple domains typically demonstrated in this age group (e.g., cognitive, motor, language, social-emotional) (6). The GSED PF items, designed to measure non-normative developmental patterns, including behavioural or regulatory challenges, are not age-ordinal and do not use the D-Score metric.

**Box 1: The Developmental score**
The Developmental score (15), or D-score, is a unidimensional latent variable measuring child development during the first three years across multiple domains. The milestones that make up the D-score conform to the Rasch model (25), thus yielding a scale with interval properties with a fixed unit (Figure 1). It is therefore possible to calculate a meaningful difference between two D-scores. Similar to height-for-age Z-score, given suitable age-conditional references, the D-score can be transformed to a Z-

[1] The full team and contributors are listed in the Acknowledgments.

For peer review only

score that accounts for children's age (i.e., Development for Age Z-score, or DAZ).

The DAZ facilitates comparisons across children of different ages.

AIMS

The primary aim of this study is to validate the GSED measures (11), through testing for measurement invariance and evaluation of the psychometric properties to measure development among children aged 0-3 years (0-36 months) globally (including creation of D-scores and Development for Age Z-score [DAZ]).

Specific Objectives:

a) Fit a Rasch model to the item data to calculate the D-scores and DAZ.
b) Investigate differential item functioning (DIF) and differential test functioning (DTF) across sites to determine measurement invariance.
c) Examine psychometric properties of the GSED SF and LF:
   - Test-retest and inter-rater reliability (score and item level),
   - Concurrent validity (association between scores on GSED and Bayley Scales of Infant and Toddler Development (Bayley-III) or Griffiths Scales of Child Development administered concurrently) (12),
   - Convergent validity (strength of association between GSED D-scores and other theoretically-relevant constructs)
   - Predictive validity (association between GSED scores six months after initial assessment).

The secondary aims are to: 1) establish preliminary reference scores[2] for optimal development on the D-score (GSED SF and LF), 2) develop and validate an adaptive

[2] The population has not been selected as a representative sample of all children aged 0-3 in each site (as would happen in a countrywide population census). Selection and recruitment of a representative sample

For peer review only - http://bmjopen.bmj.com/site/about/guidelines.xhtml

testing algorithm, and 3) obtain preliminary validity data on the psychometric properties of the GSED PF.

METHODS

Design and study sites

The GSED validation study uses a prospective cross-sectional design with a longitudinal component of age and sex stratified samples of children in seven countries. The countries are culturally, linguistically and geographically diverse, representing low-income (Bangladesh, Côte d'Ivoire, Pakistan, United Republic of Tanzania), middle-income (Brazil and The Republic of China), and high-income (The Netherlands) settings. Samples in each site are not nationally representative; however, they are diverse, e.g. covering both rural/urban settings.

Preparation and feasibility phases are described elsewhere (11), and assess feasibility of administration of GSED and associated measures including processes for translating and culturally adapting GSED and other study measures, creating data management systems, and training teams in data collection procedures.

Patient and Public involvement

Participants were involved in the study design as the burden of the assessment was discussed with them in a pilot stage through qualitative data collection. We intend to disseminate the main results to trial participants and will seek patient and public involvement in the development of an appropriate method of dissemination.

For peer review only

was beyond the scope of this study and not required for validation purposes. We are therefore developing 'reference scores' which should not be interpreted as population-sampled norms.

Study sample

The study sample includes children between 0 to 41 months of age (inclusive) living in study areas (see Table 1 for inclusion and exclusion criteria).

Table 1. Study sample inclusion and exclusion criteria

Sample	Inclusion criteria	Exclusion criteria
Total<br>per site N=1248<br>(as described in sample size section below)	1. Age 0-41 months<br>2. Family speaks to the child in same language as GSED translation<br>3. Primary caregiver available to participate	1. Missing gestational age (ultrasound or last menstrual period LMP)<br>2. Missing birth weight data<br>3. Acutely unwell at time of assessment (temporary exclusion: to be rescheduled after 7 days)

Recruitment and consent

In each site the sampling frame consists of a list of potentially eligible caregiver-child dyads residing in the defined study area. Lists of potential participants vary by site and may include: participants in local pregnancy surveillance systems, families who have previously agreed to be contacted for participation, birth registries from hospital/health centers, or families with children attending local child health/care centers. Eligible children are sampled from this list using the GSED sampling scheme (Figure 2). To minimize clustering of correlated scores within households, one child per caregiver and in multi-family household is selected, guided by age and sex quotas. For siblings or twins, one is chosen randomly. Target children's primary caregiver (person most familiar with the child and spends most time with them) is approached for consent and enrollment. A non-technically worded information sheet is shared and consent to participate is obtained at first visit. In the Netherlands, participants provide consent online, confirmed by study staff at first visit. Refusals to participate and dropouts are registered and replaced.

For peer review only

Sampling frame and schemes

Sample size for recruitment within each site is 1248 children (total 8736 children) across seven countries. After consent is provided, children are allocated by sex and age groups using a randomization procedure to one of several sampling schema (e.g., predictive, reference-score, reliability; Figure 2). See sampling Table S1 in Supplementary file S1 for sampling frame. Out of the full site sample of 1248 children, 504 children per site are randomly selected for re-evaluation 6 months later to assess predictive validity (primary aim). A second scheme indicates the minimum sub-sample of children needed to calculate preliminary reference scores (secondary aim) that will facilitate cross-country comparisons. To maximize precision of parameter estimates, larger quotas are kept for the youngest age brackets where rates of development are accelerated. A third scheme addresses inter-rater reliability for 90 children per site using two assessors who independently assess the same child sequentially or within 24 hours (12)[3]. Test-retest (intra-rater reliability) is performed by inviting 50 children per site to return for repeat assessment with the same rater within 7-10 days.

In the Netherlands, the GSED SF and PF are administered online. A subset of participants (N=32) are interviewed face-to-face to compare method of administration. To determine test-retest reliability (intra-rater reliability), the primary caregiver completes the SF and PS form online and then a second time 7-10 days later.

[3] We note that this procedure differs from typical inter-rater reliability (IRR) designs which involve simultaneous scoring of a single assessment. This sequential design was necessitated by logistical constraints. Given that this design captures both variance due to differences in raters *and* differences in occasions, the observed IRR represents a lower bound for the true inter-rater reliability of the assessments.

For peer review only - http://bmjopen.bmj.com/site/about/guidelines.xhtml

Data collection

Measures

GSED

GSED SF and LF. The creation of the GSED SF and LF is described elsewhere (10). Briefly, we constructed an item bank from previously gathered data and compiled cross-sectional and longitudinal data from 31 countries representing over 73,000 anonymized children with 109,079 assessments (using 22 established ECD instruments) (6, 13, 14). Using subject matter expert input and statistical modelling (15), we developed a caregiver-reported measure intended to capture child development at population-level (GSED SF), and a complementary direct-assessment measure to evaluate programmatic impacts (GSED LF) (10). The measures are created paper-based and app-based (GSED App) with built-in administration rules and supporting media-files (see below).

The GSED SF includes 139 items representing emerging skills and behaviors within cognitive, motor, language and social-emotional domains. All items are presented as questions to the caregiver, with binary response options (Yes/No and "Don't Know") that use start rules based on the child's age, and stop rules based on age and performance. Assessors record caregiver's responses, regardless of the assessor's observations. In the Netherlands only, the GSED SF is completed online by caregivers. The GSED SF administration includes sounds, images, and short video clips that assist in understanding, interpretating and administering the items.

The GSED LF includes 155 items capturing similar domains to the SF but, observed by the assessor following start and stop rules based on the child's age and responses. Items are organized into three grids (A, B and C) that enable assessors to measure the child's performance on similar tasks in succession, making the administration easier for both

For peer review only - http://bmjopen.bmj.com/site/about/guidelines.xhtml

assessors and children. To further facilitate administration, icons are placed next to each item that inform the assessor whether the item is observed, demonstrated to or by the child, listened for or spoken to the child. The GSED LF uses a locally constructed and low cost kit with basic materials that the child interacts with to demonstrate abilities. The kit is created by local teams with detailed guidance from WHO. Responses of all LF items are binary (skill observed/not observed).

The items in both measures are ordered by difficulty reflecting children's emerging skills. Based on the analyses from the validation, we will select the items to be included in the final GSED SF and LF versions available for use.

Psychosocial Form (PF). Unlike the SF and the LF, the GSED PF has been developed to index non-normative developmental patterns that provide a window into early manifestations of children's mental health challenges, including internalizing and externalizing behaviour problems and dysregulation (e.g., eating and sleeping). Items capturing developmentally normative information about socio-emotional competencies are included in the GSED SF and LF, as the SDG 4.2 includes children's psychosocial well-being. Because few instruments have been developed to capture psychosocial difficulties for children under 3 years, little existing data are available and the development of the GSED PF is exploratory. The PF initial prototype was created through a review of existing measures of infant and toddler mental health and consensus by subject matter experts. The GSED PF includes 47 items and reflects caregiver perceptions of the behaviors' frequency, using response options: Often; Sometimes; Never/almost never. Items are divided into two age groups: 0-6 and 6-36 months.

Contextual and demographic measures

In addition to the GSED, the validation study includes measures of children's growth and nutrition, health, environmental and contextual information (see Table 2 for measures and

For peer review only

sources). The selection of measures was based on known biological and social determinants of development (16), the demonstrated validity of the contextual measures in at least one low- and middle-income country (LMIC), and efficiency for data collection. See Supplementary File S2 for visit schedules (Tables S2a and S2b).

In three sites (Côte d'Ivoire, The Netherlands and The Republic of China) where administration of the Home Observation for Measurement of the Environment Inventory (HOME) is not feasible, household stimulation data and caregiver-child activities are collected using Family Care Indicators (FCI). In all sites, a concurrent measure of child development (Bayley-III or Griffiths Mental Development Scales) is administered in a subsample of children to determine concurrent validity of GSED to a well-established measure of the same construct.

Table 2: Study measures in addition to GSED

Construct	What the Measure Captures	Measure	Administration Mode	Time for Administer (Minutes)
Child health and household socioeconomic status (SES)	• Eligibility (exclusion - criteria)<br>• Demographic information<br>• Information about acute child health<br>• Delivery and Perinatal conditions<br>• Breastfeeding<br>• Child's health history<br>• Household socio-economic status*<br>• Caregiver education<br>• Maternal health/ chronic illness<br>• COVID-19 exposure	Eligibility and Contextual Form [Specifically developed for the study]	Caregiver Report	35
Anthropometry	• Weight at time of assessment	Anthropometry Form	Child Assessment	15

For peer review only - http://bmjopen.bmj.com/site/about/guidelines.xhtml

	• Infant Length/ Child Height at time of assessment<br>• Child's Mid-upper arm circumference at time of assessment<br>• Child's head circumference at time of assessment			
Family / home environment	• Home Environment (HOME only)<br>• Play/ Stimulation / Iinteractions between the child and other family members in the home (HOME and FCI)	Home Observation for Measurement of the Environment Inventory (HOME) (26) OR Family Care Indicators (FCI) (27) †	*HOME*: Caregiver report & Observation<br>*FCI*: Caregiver Report	*HOME*: 45<br>*FCI*: 15
	• Child neglect/abuse<br>• Exposure to violence or conflict	Childhood Psychosocial Adversity Scale (CPAS)(28) †	Caregiver Report	15
	• Family resilience	Brief Resilience Scale (BRS) (29) †	Caregiver Report	1
	• Family social support	Family Support Scale (FSS) (30) †	Caregiver Report	5
Caregiver health and well-being	• Caregiver Depressive Symptoms	The Patient Health Questionnaire-9 (PHQ-9) (31)	Caregiver Report	5
Child development	• Global child development (0-41 months)	Bayley Scales of Infant and Toddler Development (Bayley-III) (32) OR Griffiths Mental Development Scales(33) ‡	Direct child Assessment	45-60

For peer review only - http://bmjopen.bmj.com/site/about/guidelines.xhtml

	• Global child development (24-41 months)	Early Childhood Development Index 2030 (ECDI2030) (4) §	Caregiver Report	10

* Socioeconomic information on this form comes from the standard DHS multiple assets index; however, some sites have adapted the socio-economic status items to better fit their contexts

† these measures have been minorly adapted for the purpose of the study

‡ in a sub-sample (N=150)

§ in a sub-sample (all children of 24 to 41-months within the predictive validity subsamples in three countries)

Schedule

Data collection is scheduled over one to three visits depending on the study site to accommodate rules of measure administration order and location. The first administration of the GSED SF and PF is completed at home (or online in the Netherlands) to test it in the setting intended for future use (e.g., Multiple Indicator Cluster Surveys MICS or Demographic and Health Surveys DHS) and prior to administration of the GSED LF. The GSED LF is administered in a controlled environment (e.g., clinic) to match the required concurrent validity testing protocols. For the concurrent validation, the GSED and concurrent measures are administered in the same location on different days and counter-balanced in order of administration.

Training and Quality Control

Training of local master trainers is performed by the WHO team for the GSED SF, PF and LF, using slide presentations, discussion forums, audio-visual aids, and practice exercises. Local master trainers are responsible for training local field teams using materials adapted and translated to local languages. Reliable administration of the GSED measures must be met (inter-rater agreement with a master trainer of ≥ 90%) for certification.

For peer review only

360 To ensure quality assurance, 10% of all the study visits are observed by the study supervisor in person (or through video-recording in the Netherlands), covering each child age band and certified assessors. Supervisors independently complete questionnaires being administered by the assessor and complete a fidelity checklist. Assessors are given feedback based on checklist score. Supervisors review quality assurance findings with the WHO biweekly, along with discussions with the subject matter experts for further resolution, as needed.

367 The GSED application software for data collection has built-in data range and consistency checks. Data managers review and resolve issues daily in consultation with the local field team and/or WHO team.

370 **Sample size**

371 Sample size determination was based on the primary aim of assessing the psychometric properties of the GSED. To have sufficient power to estimate measurement parameters (abilities and difficulties) needed to calculate the D-score and DAZ scores at baseline and to detect DIF of 1 logit with a power of $1 - \beta = 0.90$ and a two-sided significance level of $\alpha = 0.05$, a sample of N=1248 per site is required. This sample size was calculated via optimization of the sample size at i) each age/sex stratum and ii) overall on 1000 simulated datasets generated from parameters suggested by the Rasch GSED model. See Supplementary file S1 for additional details.

379 **Statistical Analysis**

380 To construct the scores for the GSED SF and LF, a Rasch model will be fitted and the item fit statistics (infit and outfit) will be assessed. Any items with unacceptable fit levels will be removed. Items will be screened for whether they exhibit unacceptable levels of measurement non-invariance (i.e., they have approximately equal difficulties) across countries and other contextual variables. Items exhibiting unacceptable DIF (using

For peer review only - http://bmjopen.bmj.com/site/about/guidelines.xhtml

the logistic regression method) will be discarded sequentially, and the item response models will be refit using the remaining items. The expected a posteriori (EAP) method (17) will be applied to the final model to estimate the latent ability parameter (the D-score). Systematic deviations from unidimensionality will be tested by performing a principal components analysis on the residuals of the Rasch model. The ability estimates will be used to estimate preliminary developmental percentile curves against age using a Generalized Additive Model for Location Scale and Shape (GAMLSS). Following previous methodology (18) software will be written to calculate DAZ-scores based on the final dataset in R, and a user-friendly front-end version created in R (ShinyApp) and/or Excel.

Reliability (inter-rater and test-retest) for all GSED measures will be analyzed using ICC (at the score level) and Gwet's AC1 agreement (at the item level) statistics with 95% confidence intervals to determine whether items perform reliably within and between assessors (19). A cut-off value of 0.4 and above will be used to flag items as adequately reliable. Those items with agreement between 0.4 and 0.5 will be discussed to determine if modifications can be made to improve their administration and/or comprehension.

DAZ scores from the GSED SF and LF will be used to conduct validity analyses to ensure that the measures are capturing the construct they are purported to measure (construct validity). Concurrent validity will be assessed by correlating age-corrected Bayley-III or Griffiths Mental Development Scales scores with GSED DAZ scores. We anticipate that these scores will have low to moderate positive correlations. Convergent validity will be supported by statistically significant positive correlations (with 95% confidence interval) between the GSED scores and continuous contextual measures with prior evidence of association with child development. Comparisons between "known groups" will be made using the following variables: maternal education, home learning

For peer review only

opportunities, home environment, socioeconomic status (SES), maternal mental health and child anthropometry, and stunting to determine if scores discriminate between high and low categories for each variable using mean DAZ scores.

GSED scores at baseline and follow up will be correlated for predictive validity (positive association between baseline and at 6 months) and mixed-effects linear regression used to adjust for other contextual covariates and baseline scores.

Secondary (Exploratory) Aims

Reference Scores

We plan to develop a set of preliminary reference scores to facilitate comparison of DAZ scores across countries. From the full validation study sample, a sub-sample of children who have not experienced prior exposure to major known biological and environmental risk factors is selected (i.e., “reference sub-sample”) (Table 3). Such an approach relies on the assumption that the attainment of basic developmental milestones captured by the GSED of children who are free of major risk factors is relatively similar globally (20). To develop the reference scores, we will fit GAMLSS (21) to flexibly model both conditional means, conditional standard deviations of scores, and, if necessary, conditional skewness and kurtosis. We will test our assumption that the distribution of scores is equivalent across sites by adding a site indicator at each moment of the distribution, and testing site effects for their statistical significance. Where possible, we will conduct standardization of scores to assist with the interpretation of scores by pooling data across countries. We will report the corresponding parameters of the GAMLSS model at appropriate ages.

Table 3. “Reference” sub-sample exclusion criteria (healthy sub-sample)

Sample	Exclusion criteria

For peer review only

For peer review only - http://bmjopen.bmj.com/site/about/guidelines.xhtml

Minimum sub-sample of "reference" children per site N=522	1. Below secondary maternal education (<6 years of schooling)<br>2. Birthweight less than 2500 gr<br>3. Gestational age < 37 completed weeks (259 days) and ≥ 42 completed weeks (294 days) [assessed by ultrasound]<br>4. Undernutrition (weight for age, length for age, OR weight for height Z score of less than –2 on the WHO Child Growth Standards) at the time of developmental assessment<br>5. Known severe congenital birth defect<br>6. History of birth asphyxia OR neonatal sepsis requiring hospitalization<br>7. Known neurodevelopmental disorder/ disability (Severe visual problems, seizures, hearing impairment) OR other chronic health problems (that is congenital heart disease)

For peer review only

Adaptive testing

We will determine whether adaptive testing is a feasible and valid option to measure child development within the GSED (Box 2). Adaptive testing (22) is an administration method that continually adapts to the level of the child's performance, thereby reducing test administration time. Previous simulations (23) indicated that theoretically substantial gains in the precision of scores are possible when using adaptive testing even if administering fewer items.

**Box 2: Adaptive testing validation methodology**
We investigate the feasibility by applying adaptive testing in addition to the traditional "fixed" GSED administration methods in the sub-sample designated for predictive validity analyses (N=502 per site) in three sites. The adaptive test is executed using tablets that are specially programmed to continually adjust child's score after each item is administered, and to suggest the next item based on the answers already received (e.g., a more difficult item for a child with a higher score, an easier item for a child with a lower score). Once the program establishes a reliable score, the administration is

For peer review only - http://bmjopen.bmj.com/site/about/guidelines.xhtml

terminated. Both the adaptive test and the fixed test are administered with the same sub-sample during two separate visits alternating the order of administration to investigate the difference between the two modes of administration. We will investigate the following: the variance of user experience as a function of the average difficulty of milestones (leniency); the comparison of the D-score distribution under the adaptive testing procedure with the D-score distribution under the fixed GSED administration (using a z-test to assess the equivalence of the two modalities and plotting the results to show the level of concordance); and relation of the difference between the two D-scores to background variables.

Psychosocial Form

The PF measure is in an early stage and will undergo exploratory and confirmatory factor analyses to assess the internal scale structure. Associations between items and factor scores with variables suggesting a high risk of psychosocial stress, such as family resilience, social support, and family and community violence, in addition to GSED SF and LF scores (concurrent validity measures) will be examined.

ETHICS AND DISSEMINATION

The study complies with the International Ethical Guidelines for Biomedical Research Involving Human Subjects (24) and received ethical approval from the appropriate body in each site and within WHO (protocol GSED validation 004583 approved on 20.04.2020). The findings of the study will be disseminated following a comprehensive dissemination strategy to reach a diverse range of stakeholders at the local, national and international level.

DISCUSSION

For peer review only - http://bmjopen.bmj.com/site/about/guidelines.xhtml

The validation of the GSED SF and LF is a meticulous and systematic global process that introduces an innovative common metric (the D-score) that countries can use to track the progress of child development among populations of young children and to measure the impact of programmatic interventions. Additional attention is required on understanding young children's responses to psychosocial challenges within global contexts. The exploration of the GSED PF introduces an important opportunity to capture the non-normative developmental patterns among young children that are potential precursors to behaviour and psychiatric problems. The GSED validation has several important design, methodological and implementation characteristics that illustrate the rigour required to validate instruments to measure child development globally. First, it is conducted in seven countries with multiple linguistic, cultural and socioeconomic backgrounds. Second, GSED is implemented through an app-based data collection system that facilitates the implementation by reducing recording and transcribing errors and other common pitfalls of paper-based instruments. Third, this study builds on the best practices in validation by including a broad spectrum of psychometric methodologies (concurrent, predictive, convergent, and discriminant validity, test-retest and inter-rater reliability, differential item functioning, and differential test functioning). Fourth, a secondary aim builds the evidence for the creation of preliminary reference scores for the SF and LF, based on a sub-sample with minimal exposure to major biological risk factors and to the extent possible, minimal social and environmental risk factors. Fifth, we are validating an adaptive testing design that can streamline administration by tailoring and reducing the number of items required to obtain a valid score. Sixth, we are testing a new measure of young children's non-normative psychosocial development.

One notable difference between the GSED SF and LF measures and other instruments of early child development is that the GSED measures are based on a unidimensional model

For peer review only - http://bmjopen.bmj.com/site/about/guidelines.xhtml

of development through measurement approaches that are universally applicable across cultures. The measures do not follow the common multidimensional approach with separate scores for different domains or contexts. Our validation study intends to demonstrate that this model provides valid, reliable, and interpretable data globally. The GSED SF and LF may exclude some items that measure development in cultural or setting-specific ways, because the focus is on selecting items that are meaningful for understanding child development within any given setting. If specific aspects need to be captured locally, to increase cultural relevance we suggest that the GSED measures are lightly adapted with country or culture-specific item props (in agreement with WHO) and/or through the administration of additional measures.

There are several limitations to our study. Although we are validating the GSED in seven countries, including one high income setting, three sites are resource-limited (Bangladesh, Pakistan and United Republic of Tanzania). Additional evidence may be needed in high income countries to expand the validity and reliability of the GSED to population-representative samples in additional countries. Second, the GSED has been created using items that fit a Rasch model demonstrating developmental progress across ages 0-3 years (9). This univariate model makes strict assumptions designed for global population estimates and may exclude items that do not show strong age gradients or items that measure development in a culturally-specific ways. Third, GSED was developed to address population and programmatic level evaluations of early child development globally. The GSED is presently not being validated for screening or diagnosing individual children. Finally, our three secondary aims are exploratory, and will require further research, including developing global standards to replace our preliminary reference scores with more specific global norms, as in the Multi-country Growth Reference Standards for children’s weight and height. In the future we plan to

For peer review only - http://bmjopen.bmj.com/site/about/guidelines.xhtml

collect additional data from countries using strict inclusion/exclusion criteria (e.g., additional considerations around environmental risk and protective factors) to further validate our initial reference scores. Similarly, we plan to conduct further work to explore the functionality, reliability, validity, and invariance of the PF.

Conclusion

The validation of the GSED will enable countries to implement the measures with confidence. With valid, reliable, and invariant measures in hand, countries can advance initiatives to ensure that children reach their developmental potential, while reducing or eliminating disparities. Ensuring that all communities have access to policies and programs that provide nurturing care to children and families, with additional support for regions in need, promotes equity and increases the likelihood of achieving the SDGs. Once validated, the GSED measures will enable countries to adapt, modify, and evaluate their policies and programs to ensure that young children are effectively and equitably reaching their development potential and building the human capital needed for sustainable development.

For peer review only

LIST OF ABBREVIATIONS

Bayley-III	Bayley Scales of Infant and Toddler Development
BRS	Brief Resilience Scale
CI	Confidence interval
CPAS	Childhood Psychosocial Adversity Scale
DAZ	Development for Age Z-score
DHS	Demographic and Health Surveys
DIF	Differential item functioning
DTF	Differential test functioning
D-score	Developmental Score
EAP	Expected a posteriori
ECD	Early child development
ECDI 2030	Early Childhood Development Index 2030
FCI	Family Care Indicators
FSS	Family Support Scale
GAMLSS	Generalized Additive Model for Location Scale and Shape
GSED	Global Scales for Early Development
HAZ	Height-for-age z-score
HOME	Home Observation for Measurement of the Environment Inventory
ICC	Intraclass correlation coefficient
LF	Long Form
LMIC	Low- and middle-income country
MICS	Multiple Indicator Cluster Surveys
OSF	Open Science Framework
PF	Psychosocial Form
PHQ-9	The Patient Health Questionnaire-9
SDG	Sustainable Development Goals
SES	Socioeconomic status
SF	Short Form
SOPs	Standard operating procedures
WAZ	Weight-for-age z-score

For peer review only

STATEMENTS

Ethics approval: The study complies with the International Ethical Guidelines for Biomedical Research Involving Human Subjects and received ethical approval from the appropriate Ethics Review Committee (ERC) at the World Health Organization (WHO) (protocol ID GSED validation 004583 approved on 20.04.2020) and in each site.

Patient consent: Written consent is gathered by all study participants. Informed consent forms are written to be easily understood by lay persons, enabling them to understand the aims, procedures and potential risks of participation and have been approved by the WHO ERC. For participants who are illiterate, culturally acceptable options including witnessed oral consent and a thumbprint in lieu of a signature are accepted by the WHO and local ERCs.

Data sharing: Not applicable (protocol paper)

Author Contributions: All authors contributed substantively to this work. VC, TD, MG, MMB, MJ, and PK conceptualized the study protocol and drafted the manuscript; GL, GMc, DMc, MW, SvB, IE, and JS drafted the statistical analysis plan and determined the sample size calculations; AN, AR, KH, and AW drafted pieces of the manuscript. SA, AD, RA, AB, FJ, YS, IN, RK, SS, AZ, MPM, YZ, FT, ARD, AB, JZ, AH, GF, SD, NSK, FB, FJ, and MRC contributed to the adaptation of the study protocol for feasibility and on-the-ground implementation. All above authors, in addition to RK, MMP, and RN reviewed and edited the study protocol and the manuscript. All authors read and approved the final manuscript submission.

Acknowledgements: None

For peer review only

FUNDING

The authors declare that they have no competing interests.

COMPETING INTERESTS

This work was supported (alphabetical order) by Bernard van Leer Foundation, Bill & Melinda Gates Foundation, Children's Investment Fund Foundation, Jacobs Foundation and King Baudouin Foundation, United States. The funders provided financial support. The design, implementation, and writing of the manuscript were led by the World Health Organization.

For peer review only

For peer review only - http://bmjopen.bmj.com/site/about/guidelines.xhtml

REFERENCES:

1. Clark H, Coll-Seck AM, Banerjee A, Peterson S, Dalglish SL, Ameratunga S, et al. A Future for The World's Children? A WHO–UNICEF–Lancet Commission. The Lancet. 2020;395(10224):605-58.

2. UnitedNations. https://sdgs.un.org/goals/goal4 [cited 2021 November]. Department of Economic and Social Affairs, Sustainable Development].

3. Fernald LC, Prado E, Kariger P, Raikes A. A Toolkit for Measuring Early Childhood Development in Low- and Middle-Income Countries. Washington DC 20433: International Bank for Reconstruction and Development / The World Bank; 2017.

4. UNICEF. https://data.unicef.org/resources/early-childhood-development-index-2030-ecdi2030/ [updated January 2021].

5. Faruk T, King C, Muhit M, Islam MK, Jahan I, Baset KU, et al. Screening tools for early identification of children with developmental delay in low- and middle-income countries: a systematic review. BMJ Open. 2020;10(11):e038182.

6. Weber AM, Rubio-Codina M, Walker SP, Van Buuren S, Eekhout I, Grantham-McGregor SM, et al. The D-Score: A Metric for Interpreting The Early Development of Infants and Toddlers across Global Settings. BMJ Global Health. 2019;4(6):e001724.

7. McCoy DC, Waldman M, Team CF, Fink G. Measuring Early Childhood Development at a Global Scale: Evidence from the Caregiver-Reported Early Development Instruments. Early Childhood Research Quarterly. 2018;45:58-68.

8. GSEDTeam. A New Measure of Development in Children from Birth to Age 3 at Population Level: The Global Scale for Early Development (GSED). Early Childhood Matters. 2019.

9. Van Buuren S, Eekhout I, McCray G, Waldman M, Lancaster G, Black M, et al. A Scale for Tracking Child Development from Directly Observed and Caregiver-Reported Data. [Manuscript in preparation]

10. McCray G, McCoy D, Kariger P, Janus M, Black M, Chang-Lopez S, et al. The Creation of the Global Scales of Early Development (GSED) for 0-3 year old Children: Combining Subject Matter Expert judgements with big data. [Manuscript in preparation]

11. Nizar A, Kaur R, Gladstone M, Lancaster G, Baqui A, Cavallera V, et al. Assessment of Acceptability and Feasibility of the Global Scales for Early Development (GSED) Across Three Settings for 0 to 3-year-old Children. [Manuscript in preparation]

12. Streiner DL, Norman GR, Cairney J. Health measurement scales: a practical guide to their development and use: Oxford University Press, USA; 2015.

13. Lancaster GA, Kariger P, McCray G, Janus M, Gladstone M, Cavallera V, et al. Conducting a Feasibility Study in a Global Health Setting for Constructing a Caregiver-Reported Measurement Tool: An Example in Infant and Young Child Development: SAGE Publications Ltd; 2020.

14. McCoy DC, Sudfeld CR, Bellinger DC, Muhihi A, Ashery G, Weary TE, et al. Development and Validation of an Early Childhood Development Scale for Use in Low-Resourced Settings. Population Health Metrics. 2017;15(1):1-18.

15. Van Buuren S, Eekhout I. Child development with the D-score: turning milestones into measurement [version 1; peer review: 1 approved with reservations]. Gates Open Research. 2021;5(81):1-75.

For peer review only - http://bmjopen.bmj.com/site/about/guidelines.xhtml

16. Walker SP, Wachs TD, Gardner JM, Lozoff B, Wasserman GA, Pollitt E, et al. Child Development: Risk Factors for Adverse Outcomes in Developing Countries. The Lancet. 2007;369(9556):145-57.

17. Bock RD, Mislevy RJ. Adaptive EAP Estimation of Ability in a Microcomputer Environment. Applied Psychological Measurement. 1982;6(4):431-44.

18. Gladstone M, Lancaster G, McCray G, Cavallera V, Alves CR, Maliwichi L, et al. Validation of the Infant and Young Child Development (IYCD) Indicators in Three Countries: Brazil, Malawi and Pakistan. International Journal of Environmental Research and Public Health. 2021;18(11):6117.

19. Gwet KL. Computing Inter-Rater Reliability and its Variance in the Presence of High Agreement. British Journal of Mathematical and Statistical Psychology. 2008;61(1):29-48.

20. Ertem IO, Krishnamurthy V, Mulaudzi MC, Sguassero Y, Balta H, Gulumser O, et al. Similarities and Differences in Child Development from Birth to Age 3 years by Sex and Across Four Countries: A Cross-sectional, Observational Study. The Lancet Global Health. 2018;6(3):e279-e91.

21. Rigby RA, Stasinopoulos DM. Generalized Additive Models for Location, Scale and Shape. Journal of the Royal Statistical Society: Series C (Applied Statistics). 2005;54(3):507-54.

22. Wainer H, Dorans NJ, Flaugher R, Green BF, Mislevy RJ. Computerized Adaptive Testing: A Primer: Routledge; 2000.

23. Jacobusse G, Van Buuren S. Computerized Adaptive Testing for Measuring Development of Young Children. Statistics in Medicine. 2007;26(13):2629-38.

24. Singh AN. Current Status of Research Ethics and Projecting Future Initiatives. International Medical Journal. 2018;25(6).

25. Rasch G. Probabilistic Models for Some Intelligence and Attainment Tests. MESA Press, 5835 S. Kimbark Ave., Chicago: ERIC; 1993.

26. Jones PC, Pendergast LL, Schaefer BA, Rasheed M, Svensen E, Scharf R, et al. Measuring Home Environments Across Cultures: Invariance of the HOME Scale Across Eight International Sites from the MAL-ED Study. Journal of School Psychology. 2017;64:109-27.

27. Kariger P, Frongillo EA, Engle P, Britto PMR, Sywulka SM, Menon P. Indicators of Family Care for Development for Use in Multicountry Surveys. Journal of Health, Population, and Nutrition. 2012;30(4):472.

28. Berens AE, Kumar S, Tofail F, Jensen SK, Alam M, Haque R, et al. Cumulative Psychosocial Risk and Early Child Development: Validation and Use of the Childhood Psychosocial Adversity Scale in Global Health Research. Pediatric Research. 2019;86(6):766-75.

29. Smith BW, Dalen J, Wiggins K, Tooley E, Christopher P, Bernard J. The Brief Resilience Scale: Assessing the Ability to Bounce Back. International Journal of Behavioral Medicine. 2008;15(3):194-200.

30. Dunst C. The Family Support Scale: Reliability and Validity. Journal of Individual, Family, and Community Wellness. 1984;1(4):45-52.

For peer review only

31. Moriarty AS, Gilbody S, McMillan D, Manea L. Screening and Case Finding for Major Depressive Disorder Using the Patient Health Questionnaire (PHQ-9): A Meta-Analysis. General Hospital Psychiatry. 2015;37(6):567-76.

32. Bayley N. Bayley Scales of Infant and Toddler Development: Administration Manual,. 3rd edition ed. United States of America: Psychorp; 2006.

33. Jacklin L, Cockcroft K. The Griffiths Mental Developmental Scales: An Overview and a Consideration of their Relevance for South Africa. In: Laher S, Cockcroft K, editors. Psychological Assessment in South Africa. Research and applications: Wits University Press; 2013. p. 169-85.

For peer review only

For peer review only - http://bmjopen.bmj.com/site/about/guidelines.xhtml

681 **FIGURE LEGEND**

682 **Figure 1. Development chart**

683 Reproduced with permission from van Buuren S and Eekhout I (2021) (15)

684

685 **Figure 2. Study Sampling schema diagram**

For peer review only

For peer review only - http://bmjopen.bmj.com/site/about/guidelines.xhtml

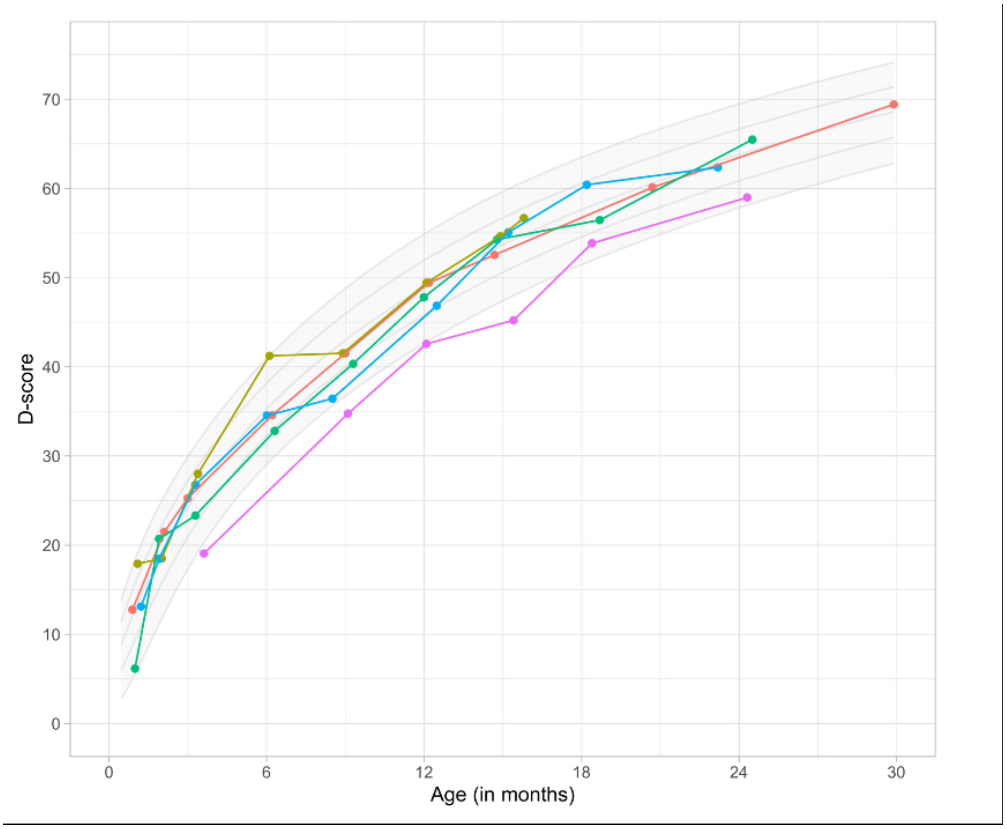

152x125mm (144 x 144 DPI)

GSED study sample

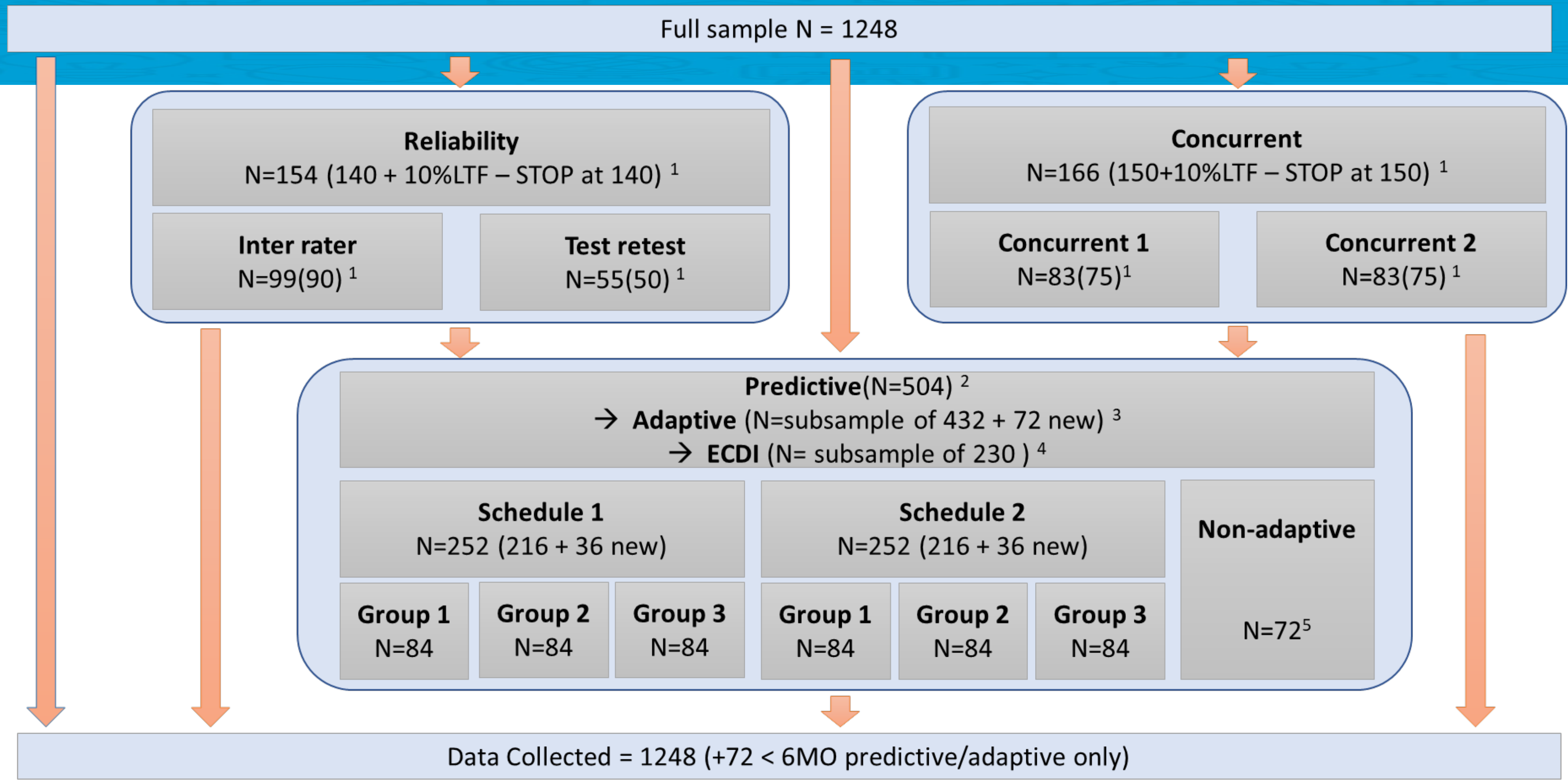

[1] The number inside parentheses is the number collected and the number outside is the number randomised to account for loss to follow-up

[2] Two additional participants have been added to the predictive to have equal numbers in each experimental group.

[3] 72 new children between 2 weeks and 6 months of age have been added to the adaptive sample to ensure coverage at the lower ages.

[4] ECDI will only be done on N=230 Children between the ages of 2+ years at the time of the predictive data collection.

[5] The 72 oldest children (36-41 months) from the predictive sample will not be part of the adaptive sample.

Supplementary file 1 – Sample size calculations and sampling frame

The sample size calculation for reliability is based on a confidence interval (CI) approach and the desired accuracy for the lower bound of the CI for the ICC estimates. In an Analysis of Variance (ANOVA) with 2-way random effects on a single score with 2 observations per subject (following Shrout and Fleiss, 1979) (27) and with a two-sided 95% confidence interval and an expected ICC of 0.875, the lower confidence interval for the inter-rater reliability sample (N=90) = 0.852. With the same parameters but an expected ICC of 0.90 the lower confidence interval for the test-retest reliability sample (N=50) is 0.830. We expect the ICC to be higher for the test-retest reliability than the inter-rater reliability as inter-rater estimates contain all the sources of error in the test-retest estimates, plus additional error between assessors (14).

To assess concurrent validity, a sample size of 150 per site produces a two-sided 95% CI 0.15-0.44, when the estimate of Pearson's product-moment correlation is 0.30, with an equal spread of participants tested across age and sex. The CI will be narrower when the data are combined across all seven countries. To assess predictive validity a sample size of 404 produces a two-sided 95% CI 0.65-0.75 when the estimate of Pearson’s product-moment correlation is 0.70 between individual scores at baseline and at the 6-month follow-up. Allowing 20% dropout at follow up, a sample size of approximately 500 participants is required.

Table S1. Sampling Frame

Sample size per site by age and sex for total population (n=1248) which includes a minimum subsample of healthy ‘reference’ children (n=522)

Age (Days)	Sex	Total Sample size	Minimum sub-sample of reference children	Predictive validity sample (6-month follow-up; age at baseline)	Reliability: Inter-rater	Reliability: Test-Retest	Concurrent validity
15-30	Male	40	20	8	2	1	4
	Female	40	20	8	2	1	2
31-61	Male	40	12	8	1	1	2
	Female	40	12	8	2	1	2
62-91	Male	40	10	8	2	1	2
	Female	40	10	8	1	0	4
92-122	Male	36	9	8	2	1	2
	Female	36	9	8	2	1	2
123-152	Male	32	8	8	1	1	2
	Female	32	8	8	2	1	2

For peer review only

153-183	Male	28	8	8	1	0	4
	Female	28	8	8	1	1	2
184-213	Male	25	7	8	2	1	2
	Female	25	7	8	1	0	2
214-244	Male	23	7	8	1	1	2
	Female	23	7	8	2	1	4
245-274	Male	21	6	8	1	1	2
	Female	21	6	8	1	1	2
275-304	Male	19	6	8	2	0	2
	Female	19	6	8	1	1	2
305-335	Male	17	6	8	1	1	4
	Female	17	6	8	2	0	2
336-365	Male	16	6	7	1	1	2
	Female	16	6	7	1	1	2
366-396	Male	14	6	7	2	1	2
	Female	14	6	7	1	1	4
397-426	Male	13	6	7	1	0	2
	Female	13	6	7	2	1	2
427-457	Male	12	5	7	1	1	2
	Female	12	5	7	1	0	2
458-487	Male	11	5	7	2	1	4
	Female	11	5	7	1	1	2
488-517	Male	11	5	7	1	1	2
	Female	11	5	7	2	1	2
518-548	Male	10	5	7	1	0	2
	Female	10	5	7	1	1	4
549-578	Male	9	5	7	2	1	2
	Female	9	5	7	1	0	2
579-609	Male	9	5	7	1	1	2
	Female	9	5	7	2	1	2
610-639	Male	9	5	7	1	1	4
	Female	9	5	7	1	1	2
640-670	Male	9	5	7	2	0	2
	Female	9	5	7	1	1	2
671-700	Male	9	5	7	1	1	2
	Female	9	5	7	2	0	4
701-730	Male	9	5	7	1	1	2
	Female	9	5	7	1	1	2
731-761	Male	9	5	7	2	1	2
	Female	9	5	7	1	1	2
762-791	Male	9	5	6	1	0	4
	Female	9	5	6	2	1	2
792-822	Male	9	5	6	1	1	2
	Female	9	5	6	1	0	2
823-852	Male	9	5	6	2	1	2
	Female	9	5	6	1	1	2

853-883	Male	9	5	6	1	1	2
	Female	9	5	6	2	1	2
884-913	Male	9	5	6	1	0	2
	Female	9	5	6	1	1	2
914-944	Male	9	5	6	2	1	2
	Female	9	5	6	1	0	2
945-974	Male	9	5	6	1	1	2
	Female	9	5	6	2	1	2
975-1004	Male	9	5	6	1	1	2
	Female	9	5	6	1	1	2
1005-1035	Male	9	5	6	2	0	2
	Female	9	5	6	1	1	2
1036-1065	Male	9	5	6	1	1	2
	Female	9	5	6	2	0	2
1066-1096	Male	9	5	6	1	1	2
	Female	9	5	6	1	1	2
1097-1126	Male	9	5	0	0	0	0
	Female	9	5	0	0	0	0
1127-1157	Male	9	5	0	0	0	0
	Female	9	5	0	0	0	0
1158-1187	Male	9	5	0	0	0	0
	Female	9	5	0	0	0	0
1188-1218	Male	9	6	0	0	0	0
	Female	9	6	0	0	0	0
1219-1248	Male	9	6	0	0	0	0
	Female	9	6	0	0	0	0
1249-1279	Male	9	7	0	0	0	0
	Female	9	7	0	0	0	0
**TOTAL**		1248	522	504	*99	**55	***166

*90 + ~10% Loss to follow up = 99; **50 + ~10% Loss to follow up = 55; ***150 + ~10% Loss to follow up = 166

For peer review only

Supplementary file 2 – Visit schedule

Table S2a.Visit Schedule for the GSED Validation Study (all sites except the Netherlands)

**Main Study Only [No Sub-sample]**	**Inter- Rater Reliability Sub- Sample**	**Test- Retest Reliability Sub- Sample**	**Concurrent Sub- Sample 1 [LF First]**	**Concurrent Sub- Sample 2 [BSID III First]**
**Visit 1 [At Home]**				
Eligibility and Consent	Eligibility and Consent	Eligibility and Consent	Eligibility and Consent	Eligibility and Consent
COVID Questionnaire	COVID Questionnaire	COVID Questionnaire	COVID Questionnaire	COVID Questionnaire
Contextual	Contextual	Contextual	Contextual	Contextual
GSED Short form [SF]	GSED Short form [SF]	GSED Short form [SF]	GSED Short form [SF]	GSED Short form [SF]
GSED Psychosocial form [PF]	GSED Psychosocial form [PF]	GSED Psychosocial form [PF]	GSED Psychosocial form [PF]	GSED Psychosocial form [PF]
HOME Inventory or Family Care Indicators (FCI)	HOME Inventory or Family Care Indicators (FCI)	HOME Inventory or Family Care Indicators (FCI)	HOME Inventory or Family Care Indicators (FCI)	HOME Inventory or Family Care Indicators (FCI)
Anthropometrics*	Anthropometrics*	Anthropometrics*	Anthropometrics*	Anthropometrics*
**Visit 2 [At home, clinic, or other setting within 48 hours of visit 1]** ***Note: For Concurrent Sample, the Visit is at the Clinic setting***				
Abbreviated Eligibility [Coversheet]	Abbreviated Eligibility [Coversheet]	Abbreviated Eligibility [Coversheet]	Abbreviated Eligibility [Coversheet]	Abbreviated Eligibility [Coversheet]
GSED Long form [LF]	GSED Long form [LF]	GSED Long form [LF]	**GSED Long form [LF]**	**BSID III**
CPAS	CPAS	CPAS	CPAS	----------------------
PHQ9	PHQ9	PHQ9	PHQ9	---------------------
Family support & Resilience Scale	Family support & Resilience Scale	Family support & Resilience Scale	Family support & Resilience Scale	----------------------
**Visit 3 [Setting and timing vary by sub-sample]**				
Visit 3 not required	Visit 3 [At home, clinic or other setting where the LF was completed- **within 24** hours of the LF]	Visit 3 [At home, clinic or other setting where the LF was completed- this should happen **7 to 10 days after** LF]	Visit 3 [Clinic setting within **24- 72 hours** of the LF- can be done at same time as Visit 2 – taking child fatigue into consideration]	Visit 3 [Clinic setting within **24- 72 hours** of the BSID III - can be done at same time as Visit 2 – taking child fatigue into consideration]
	Abbreviated Eligibility [Coversheet]	Abbreviated Eligibility [Coversheet]	Abbreviated Eligibility [Coversheet]	Abbreviated Eligibility [Coversheet]
	GSED Short form [SF]	GSED Short form [SF]	**BSID III**	**GSED Long form [LF]**
	GSED Psychosocial form [PF]	GSED Psychosocial form [PF]	----------------------	CPAS
	GSED Long form [LF]	GSED Long form [LF]	----------------------	PHQ9
	----------------------	----------------------	----------------------	Family support & Resilience Scale

* Anthropometrics may be done either at visit 1 or visit 2

Table S2b: Visit Schedule for the GSED Validation Study (the Netherlands only)

**Main Study Only [No Sub-sample]**	**Inter- Rater Reliability Sub- Sample**	**Test- Retest Reliability Sub- Sample**	**Concurrent Sub- Sample 1 [LF First]**	**Concurrent Sub- Sample 2 [BSID III First]**
**Session 1 [Online]**				
Eligibility and Consent	Eligibility and Consent	Eligibility and Consent	Eligibility and Consent	Eligibility and Consent
Contextual	Contextual	Contextual	Contextual	Contextual
GSED Short form [SF]	GSED Short form [SF]	GSED Short form [SF]	GSED Short form [SF]	GSED Short form [SF]
GSED Psychosocial scale [PS]	GSED Psychosocial scale [PS]	GSED Psychosocial scale [PS]	GSED Psychosocial scale [PS]	GSED Psychosocial scale [PS]
**Visit 1 [At clinic within 48 hours of session1]**				
Abbreviated Eligibility [Coversheet]	Abbreviated Eligibility [Coversheet]	Abbreviated Eligibility [Coversheet]	Abbreviated Eligibility [Coversheet]	Abbreviated Eligibility [Coversheet]
GSED Long form [LF]	GSED Long form [LF]	GSED Long form [LF]	**GSED Long form [LF]**	**BSID III**
Anthropometrics	Anthropometrics	Anthropometrics	Anthropometrics	Anthropometrics
**Session 2 [Online, Test-Retest of SF/PSY within 7 to 10 days of online session 1]**				
Abbreviated Eligibility [Coversheet]	Abbreviated Eligibility [Coversheet]	Abbreviated Eligibility [Coversheet]	Abbreviated Eligibility [Coversheet]	Abbreviated Eligibility [Coversheet]
COVID Questionnaire	COVID Questionnaire	COVID Questionnaire	COVID Questionnaire	COVID Questionnaire
----------------------	----------------------	GSED Short form [SF]	----------------------	----------------------
----------------------	----------------------	GSED Psychosocial scale [PS]	----------------------	----------------------
CPAS	CPAS	CPAS	CPAS	CPAS
PHQ9	PHQ9	PHQ9	PHQ9	PHQ9
Family support & Resilience Scale	Family support & Resilience Scale	Family support & Resilience Scale	Family support & Resilience Scale	Family support & Resilience Scale
Family Care Indicators (FCI)	Family Care Indicators (FCI)	Family Care Indicators (FCI)	Family Care Indicators (FCI)	Family Care Indicators (FCI)
**Visit 2 [At clinic, timing varies by sub-sample]**				
Visit 2 not required	Visit 2 [**within 24** hours of the LF]	Visit 2 [**7 to 10 days after** LF]	Visit 2 [within **24- 72 hours** of the LF- can be done at same time as Visit 1 – taking child fatigue into consideration]	Visit 2 [within **24- 72 hours** of the BSID III - can be done at same time as Visit 1 – taking child fatigue into consideration]
	Abbreviated Eligibility [Coversheet]	Abbreviated Eligibility [Coversheet]	Abbreviated Eligibility [Coversheet]	Abbreviated Eligibility [Coversheet]
	GSED Long form [LF]	GSED Long form [LF]	**BSID III**	**GSED Long form [LF]**

BMJ Open

Protocol for Validation of the Global Scales for Early Development (GSED) for Children under 3 Years of Age in Seven Countries

Journal:	*BMJ Open*
Manuscript ID	bmjopen-2022-062562.R1
Article Type:	Protocol
Date Submitted by the Author:	23-Sep-2022

Complete List of Authors:	Cavallera, Vanessa; World Health Organization, Department of Mental Health and Substance Use<br>Lancaster, Gillian; Keele University School of Medicine<br>Gladstone, Melissa; University of Liverpool Institute of Translational Medicine, Women and Children's Health<br>Black, Maureen; RTI International; University of Maryland School of Medicine<br>McCray, Gareth; Keele University School of Medicine<br>Nizar, Ambreen; The Aga Khan University, Department of Peadiatrics<br>Ahmed, Salahuddin; Projahnmo Research Foundation<br>Dutta, Arup; Center for Public Health Kinetics<br>Anago, Romuald ; Innovations for Poverty Action<br>Brentani, Alexandra; University of Sao Paulo, Pediatrics<br>Jiang, Fan; Shanghai Children's Medical Center Affiliated to Shanghai Jiaotong University School of Medicine<br>Schönbeck, Yvonne; Netherlands Organization for Applied Scientific Research, Department of Child Health<br>McCoy, Dana; Harvard Graduate School of Education<br>Kariger, Patricia; University of California Berkeley School of Public Health<br>Weber, Ann; University of Nevada Reno<br>Raikes, Abbie; University of Nebraska Medical Center College of Public Health<br>Waldman, Marcus; University of Nebraska Medical Center College of Public Health<br>van Buuren, Stef; TNO, Department of Statistics<br>Kaur, Raghbir; World Health Organization<br>Maillard, Pérez; World Health Organization<br>Nisar, Muhammad; The Aga Khan University, Pediatrics and Child Health<br>Khanam, Rasheda; Johns Hopkins University, International Center for Maternal and Newborn Health, Department of International Health, Johns Hopkins Bloomberg School of Public Health<br>Sazawal, Sunil ; Centre for Public Health Kinetics<br>Zongo, Arsène; Innovations for Poverty Action<br>Pacifico Mercadante, Mariana; University of Sao Paulo, Pediatrics<br>Zhang, Yunting ; Shanghai Children's Medical Center Affiliated to Shanghai Jiaotong University School of Medicine<br>Roy, Arunangshu ; Projahnmo Research Foundation<br>Hepworth, Katelyn; University of Nebraska-Lincoln College of Education and Human Sciences

	Fink, Günther ; Schweizerisches Tropen- und Public Health-Institut, Household Economics and Health System Research Unit<br>Rubio-Codina, Marta; Inter-American Development Bank<br>Tofail, Fahmida; International Centre for Diarrhoeal Disease Research Bangladesh, Centre for Nutrition & Food Security<br>Eekhout, Iris; TNO, Child Health<br>Seiden, Jonathan; Harvard Graduate School of Education<br>Norton, Rebecca; World Health Organization<br>Baqui, Abdullah; Johns Hopkins University Bloomberg School of Public Health<br>Zhao, Jin; Shanghai Children's Medical Center Affiliated to Shanghai Jiaotong University School of Medicine<br>Holzinger, Andreas; Innovations for Poverty Action<br>Detmar, Symone; TNO<br>Kembou, Samuel; Innovations for Poverty Action<br>Begum, Farzana; The Aga Khan University, Department of Peadiatrics<br>Jehan, Fyezah; The Aga Khan University, Paediatrics and Child Health<br>Dua, Tarun; World Health Organization<br>Janus, Magdalena; McMaster University, Offord Centre for Child Studies

**Primary Subject Heading**:	Public health
Secondary Subject Heading:	Paediatrics
Keywords:	MENTAL HEALTH, Paediatric neurology < NEUROLOGY, Developmental neurology & neurodisability < PAEDIATRICS, PUBLIC HEALTH, EPIDEMIOLOGY, International health services < HEALTH SERVICES ADMINISTRATION & MANAGEMENT

SCHOLARONE™
Manuscripts

BMJ Open

Title: Protocol for Validation of the Global Scales for Early Development (GSED) for Children under 3 Years of Age in Seven Countries

Authors: Cavallera V, Lancaster G, Gladstone M, Black MM, McCray G, Nizar A, Ahmed S, Dutta A, Anago R, Brentani A, Jiang F, Schönbeck Y, McCoy D, Kariger P, Weber A, Raikes A, Waldman M, van Buuren S, Kaur R, Pérez Maillard M, Nisar I, Khanam R, Sazawal S, Zongo A, Pacifico Mercadante M, Zhang Y, Roy AD, Hepworth K, Fink G, Rubio Codina M, Tofail F, Eekhout I, Seiden J, Norton R, Baqui AH, Zhao J, Holzinger A, Detmar S, Kembou S N, Begum F, Jehan F, Dua T, Janus M.

Affiliations:

Corresponding author: Dr Vanessa Cavallera, MD, MPH, World Health Organization (WHO), Brain Health Unit in Department of Mental Health and Substance Use, Av. Appia 20, 1202 Geneva, Switzerland. cavallerav@who.int

Prof Gillian Lancaster, PhD, MSc, BSc (Hons), Clinical Trials Unit, School of Medicine, Keele University, UK

Prof Melissa Gladstone, MD, International Child Health and Neurodevelopmental Paediatrics, Department of Women and Children's Health, Institute of Translational Medicine, University of Liverpool, Alder Hey Children's NHS Foundation Trust, UK.

Prof Maureen Black, PhD, Department of Pediatrics, University of Maryland School of Medicine, Baltimore, MD, USA and RTI International, Research Park, NC, USA

Dr Gareth McCray, MA, MRes, PhD, Research Associate, the School of Medicine, Keele University, Keele, UK.

Ambreen Nizar, BDS, MSc, Department of Peadiatrics, Aga Khan University, Karachi Pakistan.

For peer review only

Salahuddin Ahmed, MBBS, Projahnmo Research Foundation, Dhaka, Bangladesh

Arup Dutta, MBA, Center for Public Health Kinetics, New Delhi, India

Romuald Kouadio E Anago, MA, BA, Innovations for Poverty Action (IPA), Abidjan, Côte d'Ivoire

Dr. Alexandra Valeria Maria Brentani, PhD, Dept. of Pediatrics, University of Sao Paulo Medical School, Brazil

Prof. Fan Jiang, MD, PhD, Department of Developmental and Behavioral Pediatrics, National Children's Medical Center, Shanghai Children's Medical Center, affiliated to School of Medicine Shanghai Jiao Tong University, China

Dr. Yvonne Schönbeck, PhD, Msc, Dept. Child Health, Netherlands Organization for Applied Scientific Research (TNO), Leiden, The Netherlands

Dr Dana McCoy, PhD, Harvard Graduate School of Education, Cambridge, MA, USA

Dr Patricia Kariger, PhD CEGA (Center for Effective Global Action), School of Public Health, University of California, Berkeley, California, USA.

Dr Ann Weber, PhD, MPH, School of Public Health, University of Nevada, Reno, NV, USA

Dr Hilary Abigail Raikes, MPH, PhD, University of Nebraska Medical Center, College of Public Health, Omaha, NE, USA

Dr. Marcus Waldman, PhD, University of Nebraska Medical Center, College of Public Health, Omaha, NE, USA

Prof Stef van Buuren, PhD, Department of Child Health, Netherlands Organization for Applied Scientific Research TNO, Leiden, The Netherlands

For peer review only

Raghbir Kaur, MPH, DMD, MS, Consultant World Health Organization (WHO), Brain Health Unit in Department of Mental Health and Substance Use, Geneva, Switzerland

Michelle Pérez Maillard, MSc, Consultant World Health Organization (WHO), Brain Health Unit in Department of Mental Health and Substance Use, Geneva, Switzerland

Prof. Muhammad Imran Nisar, MBBS, MSc, Department of Peadiatrics, Aga Khan University, Karachi Pakistan.

Dr. Rasheda Khanam, MBBS, MPH, PhD, International Center for Maternal and Newborn Health, Department of International Health, Johns Hopkins University Bloomberg School of Public Health, Baltimore, MD, USA

Dr. Sunil Sazawal, MBBS, MPH, PhD, Center for Public Health Kinetics New Delhi, India

Mr. Arsène Zongo, MA, BA, Innovations for Poverty Action (IPA), Abidjan, Côte d'Ivoire

Dr. Mariana Pacifico Mercadante, MD, Dept. of Pediatrics, University of Sao Paulo Medical School, Brazil

Dr. Yunting Zhang, PhD, Child Health Advocacy Institute, National Children's Medical Center, Shanghai Children's Medical Center, affiliated to School of Medicine Shanghai Jiao Tong University, China

Dr. Arunangshu D. Roy, MBBS, Projahnmo Research Foundation, Dhaka, Bangladesh

Katelyn Hepworth, MA, University of Nebraska-Lincoln, College of Education and Human Sciences, NE, USA

Prof Günther Fink, PhD, Swiss Tropical and Public Health Institute, Basel, Switzerland

For peer review only

Dr Marta Rubio-Codina, PhD, Inter-American Development Bank, Washington, D.C., USA

Dr. Fahmida Tofail, MBBS, PhD, International Centre for Diarrhoeal Disease Research Icddr,b, Dhaka, Bangladesh

Dr. Iris Eekhout, PhD, MSc, Department of Child Health, Netherlands Organization for Applied Scientific Research TNO, Leiden, The Netherlands

Jonathan Seiden, EdM, Harvard Graduate School of Education, Cambridge, MA, USA

Rebecca Norton, Consultant World Health Organization (WHO), Brain Health Unit in Department of Mental Health and Substance Use, Geneva, Switzerland

Dr. Abdullah H. Baqui, MBBS, DrPH, Johns Hopkins Bloomberg School of Public Health, Baltimore, Maryland, USA

Dr. Jin Zhao, MD, PhD, Department of Developmental and Behavioral Pediatrics, National Children's Medical Center, Shanghai Children's Medical Center, affiliated to School of Medicine Shanghai Jiao Tong University, China

Andreas Holzinger, MA, BA, Innovations for Poverty Action (IPA), Abidjan, Côte d'Ivoire

Dr. Symone Detmar, PhD, Msc, Department of Child Health, Netherlands Organization for Applied Scientific Research TNO, Leiden, The Netherlands

Dr, Samuel Nzale Kembou, PhD, MA, BA, Innovations for Poverty Action (IPA), Abidjan, Côte d'Ivoire

Farzana Begum, MSc, Department of Peadiatrics, Aga Khan University, Karachi Pakistan.

For peer review only

Prof. Fyezah Jehan, MBBS, FCPS, MSc, Department of Peadiatrics, Aga Khan University, Karachi Pakistan.

Dr Tarun Dua, MD, World Health Organization (WHO), Brain Health Unit in Department of Mental Health and Substance Use, Geneva, Switzerland

Prof Magdalena Janus, MBBS, FCPS, MSc, Offord Centre for Child Studies, McMaster University, Hamilton ON, Canada.

Disclaimers:

The author is a member of the World Health Organization. The author alone is responsible for the views expressed in this publication and they do not necessarily represent the decisions, policy or views of the World Health Organization. [Applies to Cavallera V, Dua T, Kaur R, Pérez Maillard M and Norton R]

The views here presented do not represent the Inter-American Development Bank, its board of directors, or the countries it represents. [Applies to Rubio Codina M]

Wordcount: 4000

Keywords: Early childhood development, global, measurement, index, population, evaluation, validation, protocol, programmes.

For peer review only

ABSTRACT

Introduction. Children's early development is affected by caregiving experiences, with life-long health and wellbeing implications. Governments and civil societies need population-based measures to monitor children's early development and ensure that children receive the care needed to thrive. To this end, the World Health Organization (WHO) developed the Global Scales for Early Development (GSED) to measure children's early development (ages 0-3 years). The GSED includes three measures: 1) short form (SF) for population-evaluation (caregiver-report), 2) complementary long form (LF) for programmatic-evaluation (direct assessment), and 3) psychosocial form (PF) for psychosocial development evaluation (caregiver-report). The primary aim of this protocol is to validate the GSED SF and LF. Secondary aims are to create preliminary reference scores for the GSED SF and LF, validate an adaptive testing algorithm, and assess the feasibility and preliminary validity of the GSED PF.

Methods and Analysis. We will conduct the validation in seven countries (Bangladesh, Brazil, Côte d'Ivoire, Pakistan, The Netherlands, The Republic of China, United Republic of Tanzania), varying in geography, language, culture and income through a one-year prospective design, combining cross-sectional and longitudinal methods with 1248 children per site, stratified by age and sex. The GSED generates an innovative common metric (Development-score: D-score) using the Rasch model and a development-for-age z-score (DAZ). We will evaluate six psychometric properties of the GSED SF and LF: concurrent validity, predictive validity at six months, convergent and discriminant validity, and test-retest and inter-rater reliability. We will evaluate measurement invariance by comparing differential item functioning (DIF) and differential test functioning (DTF) across sites.

For peer review only - http://bmjopen.bmj.com/site/about/guidelines.xhtml

Ethics and dissemination. This study has received ethical approval from the WHO (protocol GSED validation 004583 20.04.2020) and approval in each site . Study results will be disseminated through webinars and publications from WHO, international organisations, academic journals, and conference proceedings.

Registration details: Open Science Framework (OSF) https://osf.io/ on 19/11/2021 (DOI 10.17605/OSF.IO/KX5T7; identifier: osf-registrations-kx5t7-v1)

ARTICLE SUMMARY

Strengths and limitations of this study

- The study collects validation data (n = 8736 children) for the Global Scales for Early Development (GSED) in seven countries that vary in geographic, linguistic, cultural and sociodemographic characteristics.
- The methods for the validation of GSED are systematic across sites and follow rigorous standard operating procedures based on the best scientific evidence available.
- A tablet-based App is used for data collection to make the administration of the GSED measures user-friendly, to reduce recording and transcribing errors, and to facilitate adaptive testing.
- The GSED SF and LF aims to include items that are culturally neutral and fit the Rasch model, which assumes that child development milestones are age-ordinal, to create D-scores while psychosocial items are included in a separate measure (GSED psychosocial form [PF]) and cultural-specific items can be supplemented by countries.
- The three secondary aims (preliminary reference scores, an adaptive testing algorithm, and the feasibility and validity of the GSED PF), are exploratory and will require further research.

For peer review only - http://bmjopen.bmj.com/site/about/guidelines.xhtml

INTRODUCTION

Prenatal and early postnatal experiences have significant impacts on early childhood development (ECD) and can influence the accrual of health, well-being, and productivity throughout the life-course (1). To promote current and sustainable peace and prosperity, the United Nations has focused the Sustainable Development Goals (SDG) on improving children's outcomes in the early years through multiple targets. The most explicit target for young children is SDG 4 (Education goal), which requires reporting on the "proportion of children under 5 years of age who are developmentally on track in health, learning and psychosocial well-being, by sex" (2).

There are few valid measures that can be used globally to assess child development for children under three years of age. Current measures of ECD range from proxy measures (e.g., prevalence of country-level stunting and poverty) to detailed measures of individual performance on developmental tasks (3). The Early Childhood Development Index 2030 (ECDI 2030) (4) does not include children below two years of age. A recent review has identified the creation and validation of population-based instruments for assessing very young children as a global priority (5).

The Global Scales for Early Development (GSED) build on advances made by analyses of existing global datasets (6), and new data collection (7) that demonstrated the cross cultural applicability of items that measure young children's development. Three research teams (8) joined efforts to develop the GSED in response to the pressing need for instruments and metrics to measure ECD at population and programmatic levels across diverse parts of the world.

For peer review only

180 **The Global Scales for Early Development (GSED)**

181 The GSED consist of three open-access measures developed by a WHO-led team[1] to provide a standardized methodology for measuring the development of children aged 0-3 years (0-36 months) across diverse cultures and contexts (9, 10). They are developed for three objectives: 1) for population-level evaluation based on caregiver-report, GSED Short Form (SF); 2) for programmatic evaluation in combination with SF, direct child assessment, GSED Long Form (LF); and 3) for measuring psychosocial behaviours, caregiver-reported GSED Psychosocial Form (PF). The development and piloting of the GSED SF, LF, and PF are described elsewhere (9).

189 The GSED SF and LF produce metrics on the same age-ordinal scale and quantify the same latent construct. The Developmental Score (D-score) (see Box 1) underlies both measures and reflects children's overall development across multiple domains typically demonstrated in this age group (e.g., cognitive, motor, language, social-emotional) (6).

193 The GSED PF items, designed to measure non-normative developmental patterns, including behavioural or regulatory challenges, are not age-ordinal and do not use the D-Score metric.

**Box 1: The Developmental score**
The Developmental score (11), or D-score, is a unidimensional latent variable measuring child development during the first three years across multiple domains. The milestones that make up the D-score conform to the Rasch model (12), thus yielding a scale with interval properties with a fixed unit (Figure 1). It is therefore possible to calculate a meaningful difference between two D-scores. Similar to height-for-age Z-score, given suitable age-conditional references, the D-score can be transformed to a Z-

[1] The full team and contributors are listed in the Acknowledgments.

For peer review only - http://bmjopen.bmj.com/site/about/guidelines.xhtml

score that accounts for children's age (i.e., Development for Age Z-score, or DAZ). The DAZ facilitates comparisons across children of different ages.

196 **AIMS**

197 The primary aim of this study is to validate the GSED measures (13), through testing for measurement invariance and evaluation of the psychometric properties to measure development among children aged 0-3 years (0-36 months) globally (including creation of D-scores and Development for Age Z-score [DAZ]).

201 Specific Objectives:

202 a) Fit a Rasch model to the item data to calculate the D-scores and DAZ.

203 b) Investigate differential item functioning (DIF) and differential test functioning (DTF) across sites to determine measurement invariance.

205 c) Examine psychometric properties of the GSED SF and LF:

206 - Test-retest and inter-rater reliability (score and item level),

207 - Concurrent validity (association between scores on GSED and Bayley Scales of Infant and Toddler Development (Bayley-III) or Griffiths Scales of Child Development administered concurrently) (14),

210 - Convergent validity (strength of association between GSED D-scores and other theoretically-relevant constructs)

212 - Predictive validity (association between GSED scores six months after initial assessment).

214 The secondary aims are to: 1) establish preliminary reference scores[2] for optimal development on the D-score (GSED SF and LF), 2) develop and validate an adaptive

[2] The population has not been selected as a representative sample of all children aged 0-3 in each site (as would happen in a countrywide population census). Selection and recruitment of a representative sample

testing algorithm, and 3) obtain preliminary validity data on the psychometric properties of the GSED PF.

METHODS

Design and study sites

The GSED validation study uses a prospective cross-sectional design with a longitudinal component of age and sex stratified samples of children in seven countries. The countries are culturally, linguistically and geographically diverse, representing low-income (Bangladesh, Côte d'Ivoire, Pakistan, United Republic of Tanzania), middle-income (Brazil and The Republic of China), and high-income (The Netherlands) settings. Samples in each site are not nationally representative; however, they are diverse, e.g. covering both rural/urban settings.

Preparation and feasibility phases are described elsewhere (13), and assess feasibility of administration of GSED and associated measures including processes for translating and culturally adapting GSED and other study measures, creating data management systems, and training teams in data collection procedures.

Patient and Public involvement

Caregivers of children 0-41 month-olds were involved in the study design as the burden of the assessment was discussed with them in a pilot stage through qualitative data collection. We intend to disseminate the main results to trial participants and will seek patient and public involvement in the development of an appropriate method of dissemination.

For peer review only

---

was beyond the scope of this study and not required for validation purposes. We are therefore developing 'reference scores' which should not be interpreted as population-sampled norms.

Study sample

The study sample includes children between 0 to 41 months of age (inclusive) living in study areas (see Table 1 for inclusion and exclusion criteria). The small sample of children from 36-41 months aims to ensure that parameters are estimated with adequate precision for children at the top of our age range (36 months).

Table 1. Study sample inclusion and exclusion criteria

Sample	Inclusion criteria	Exclusion criteria
Total per site N=1248 (as described in sample size section below)	1. Age 0-41 months<br>2. Family speaks to the child in same language as GSED translation<br>3. Primary caregiver available to participate	1. Missing gestational age (ultrasound or last menstrual period LMP)<br>2. Missing birth weight data<br>3. Acutely unwell at time of assessment (temporary exclusion: to be rescheduled after 7 days)

Recruitment and consent

In each site the sampling frame consists of a list of potentially eligible caregiver-child dyads residing in the defined study area. Lists of potential participants are created in compliance with ethical review boards approved processes; they vary by site and may include: participants in local pregnancy surveillance systems, families who have previously agreed to be contacted for participation, from hospital/health center registries, or families with children attending local child health/care centers. Sites using registries will rely on hospital or health center staff (unaffiliated with GSED) to contact families and obtain consent for sharing their information with the GSED team. A sample listing of the pre-consented families will be provided to the GSDE team for recruitment. Sites recruiting families from local child health/care centers will rely on advertisements or flyers with information about the project, participation requirements, GSED team contact

For peer review only - http://bmjopen.bmj.com/site/about/guidelines.xhtml

information for questions, and a scan code or website link for interested families to provide basic eligibility information and consent to be contacted for enrollment. Eligible children are sampled from this list using the GSED sampling scheme (Figure 2). To minimize clustering of correlated scores within households, one child per caregiver and in multi-family household is selected, guided by age and sex quotas. For siblings or twins, one is chosen randomly. Target children's primary caregiver (person most familiar with the child and spends most time with them) is approached for consent and enrollment. A non-technically worded information sheet is shared and consent to participate is obtained at first visit. In the Netherlands, participants provide consent online, confirmed by study staff at first visit. Refusals to participate and dropouts are registered and replaced.

Sampling frame and schemes

Sample size for recruitment within each site is 1248 children (total 8736 children) across seven countries. After consent is provided, children are allocated by sex and age groups using a randomization procedure to one of several sampling schema (e.g., predictive, reference-score, reliability; Figure 2). See sampling Table S1 in Supplementary file S1 for sampling frame. Out of the full site sample of 1248 children, 504 children per site are randomly selected for re-evaluation 6 months later to assess predictive validity (primary aim). A second scheme indicates the minimum sub-sample of children needed to calculate preliminary reference scores (secondary aim) that will facilitate cross-country comparisons. To maximize precision of parameter estimates, larger quotas are kept for the youngest age brackets where rates of development are accelerated. A third scheme addresses inter-rater reliability for 90 children per site using two assessors who

For peer review only

279 independently assess the same child sequentially or within 24 hours (14)[3]. Test-retest (intra-rater reliability) is performed by inviting 50 children per site to return for repeat assessment with the same rater within 7-10 days. For concurrent validity, to assess the GSED against the Bayley-III, a sample size of N = 150 per country produces a two-sided 95% confidence interval 0.15-0.44, when the estimate of Pearson's product-moment correlation is 0.3, with an equal spread of participants tested across age and sex.

In the Netherlands, the GSED SF and PF are administered online. A subset of participants (N=32) are interviewed face-to-face to compare method of administration. To determine test-retest reliability (intra-rater reliability), the primary caregiver completes the SF and PS form online and then a second time 7-10 days later.

Data collection

Measures

GSED

GSED SF and LF. The creation of the GSED SF and LF is described elsewhere (10). Briefly, we constructed an item bank from previously gathered data and compiled cross-sectional and longitudinal data from 31 countries representing over 73,000 anonymized children with 109,079 assessments (using 22 established ECD instruments) (6, 15, 16). Using subject matter expert input and statistical modelling (11), we developed a caregiver-reported measure intended to capture child development at population-level (GSED SF), and a complementary direct-assessment measure to evaluate programmatic

[3] We note that this procedure differs from typical inter-rater reliability (IRR) designs which involve simultaneous scoring of a single assessment. This sequential design was necessitated by logistical constraints. Given that this design captures both variance due to differences in raters *and* differences in occasions, the observed IRR represents a lower bound for the true inter-rater reliability of the assessments.

For peer review only - http://bmjopen.bmj.com/site/about/guidelines.xhtml

impacts (GSED LF) (10). The measures are created paper-based and app-based (GSED App) with built-in administration rules and supporting media-files (see below).

The GSED SF includes 139 items representing emerging skills and behaviors within cognitive, motor, language and social-emotional domains. All items are presented as questions to the caregiver, with binary response options (Yes/No and "Don't Know") that use start rules based on the child's age, and stop rules based on age and performance. Assessors record caregiver's responses, regardless of the assessor's observations. In the Netherlands only, the GSED SF is completed online by caregivers. The GSED SF administration includes sounds, images, and short video clips that assist in understanding, interpretating and administering the items.

The GSED LF includes 155 items capturing similar domains to the SF but, observed by the assessor following start and stop rules based on the child's age and responses. LF items must either be observed incidentally or by eliciting the behaviour or both, depending on the item. Items are organized into three grids (A, B and C) that enable assessors to measure the child's performance on similar tasks in succession, making the administration easier for both assessors and children. To further facilitate administration, icons are placed next to each item that inform the assessor whether the item is observed, demonstrated to or by the child, listened for or spoken to the child. The GSED LF uses a locally constructed and low cost kit with basic materials that the child interacts with to demonstrate abilities. The kit is created by local teams with detailed guidance from WHO. Responses of all LF items are binary (skill observed/not observed).

The items in both measures are ordered by difficulty reflecting children's emerging skills. Based on the analyses from the validation, we will select the items to be included in the final GSED SF and LF versions available for use.

For peer review only

Psychosocial Form (PF). Unlike the SF and the LF, the GSED PF has been developed to index non-normative developmental patterns that provide a window into early manifestations of children's mental health challenges, including internalizing and externalizing behaviour problems and dysregulation (e.g., eating and sleeping). Items capturing developmentally normative information about socio-emotional competencies are included in the GSED SF and LF, as the SDG 4.2 includes children's psychosocial well-being. Because few instruments have been developed to capture psychosocial difficulties for children under 3 years, little existing data are available and the development of the GSED PF is exploratory. The PF initial prototype was created through a review of existing measures of infant and toddler mental health and consensus by subject matter experts. The GSED PF includes 47 items and reflects caregiver perceptions of the behaviors' frequency, using response options: Often; Sometimes; Never/almost never. Items are divided into two age groups: 0 to <6 and 6 to <36 months.

Contextual and demographic measures

In addition to the GSED, the validation study includes measures of children's growth and nutrition, health, environmental and contextual information (see Table 2 for measures and sources). The selection of measures was based on known biological and social determinants of development (17), the demonstrated validity of the contextual measures in at least one low- and middle-income country (LMIC), and efficiency for data collection. See Supplementary File S2 for visit schedules (Tables S2a and S2b).

In three sites (Côte d'Ivoire, The Netherlands and The Republic of China) where administration of the Home Observation for Measurement of the Environment Inventory (HOME) is not feasible, household stimulation data and caregiver-child activities are collected using Family Care Indicators (FCI). In all sites, a concurrent measure of child development (Bayley-III or Griffiths Mental Development Scales) is administered in a

For peer review only

For peer review only - http://bmjopen.bmj.com/site/about/guidelines.xhtml

348 subsample of children to determine concurrent validity of GSED to a well-established
349 measure of the same construct.

350 **Table 2: Study measures in addition to GSED**

Construct	What the Measure Captures	Measure	Administration Mode	Time for Administer (Minutes)
Child health and household socioeconomic status (SES)	• Eligibility (exclusion - criteria)<br>• Demographic information<br>• Information about acute child health<br>• Delivery and Perinatal conditions<br>• Breastfeeding<br>• Child's health history<br>• Household socio-economic status*<br>• Caregiver education<br>• Maternal health/ chronic illness<br>• COVID-19 exposure	Eligibility and Contextual Form [Specifically developed for the study]	Caregiver Report	35
Anthropometry	• Weight at time of assessment<br>• Infant Length/ Child Height at time of assessment<br>• Child's Mid-upper arm circumference at time of assessment<br>• Child's head circumference at time of assessment	Anthropometry Form	Child Assessment	15
Family / home environment	• Home Environment (HOME only)<br>• Play/ Stimulation / Iinteractions between the child and other family members in the	Home Observation for Measurement of the Environment Inventory (HOME) (18) OR Family Care Indicators (FCI) (19) †	*HOME*: Caregiver report & Observation<br>*FCI*: Caregiver Report	*HOME*: 45<br>*FCI*: 15

For peer review only - http://bmjopen.bmj.com/site/about/guidelines.xhtml

	home (HOME and FCI)			
	• Child neglect/abuse<br>• Exposure to violence or conflict	Childhood Psychosocial Adversity Scale (CPAS)(20) [†]	Caregiver Report	15
	• Family resilience	Brief Resilience Scale (BRS) (21) [†]	Caregiver Report	1
	• Family social support	Family Support Scale (FSS) (22) [†]	Caregiver Report	5
Caregiver health and well-being	• Caregiver Depressive Symptoms	The Patient Health Questionnaire-9 (PHQ-9) (23)	Caregiver Report	5
Child development	• Global child development (0-41 months)	Bayley Scales of Infant and Toddler Development (Bayley-III) (24) OR Griffiths Mental Development Scales(25) [‡]	Direct child Assessment	45-60
	• Global child development (24-41 months)	Early Childhood Development Index 2030 (ECDI2030) (4) [§]	Caregiver Report	10

* Socioeconomic information on this form comes from the standard DHS multiple assets index; however, some sites have adapted the socio-economic status items to better fit their contexts

[†] these measures have been minorly adapted for the purpose of the study

[‡] in a sub-sample (N=150)

[§] in a sub-sample (all children of 24 to 41-months within the predictive validity subsamples in three countries)

Schedule

Data collection is scheduled over one to three visits depending on the study site to accommodate rules of measure administration order and location. The first

For peer review only

administration of the GSED SF and PF is completed at home (or online in the Netherlands) to test it in the setting intended for future use (e.g., Multiple Indicator Cluster Surveys MICS or Demographic and Health Surveys DHS) and prior to administration of the GSED LF. The GSED LF is administered in a controlled environment (e.g., clinic) to match the required concurrent validity testing protocols. For the concurrent validation, the GSED and concurrent measures are administered in the same location on different days and counter-balanced in order of administration.

Training and Quality Control

Training of local master trainers is performed by the WHO team for the GSED SF, PF and LF, using slide presentations, discussion forums, audio-visual aids, and practice exercises. Local master trainers are responsible for training local field teams using materials adapted and translated to local languages. Reliable administration of the GSED measures must be met (inter-rater agreement with a master trainer of ≥ 90%) for certification.

To ensure quality assurance, 10% of all the study visits are observed by the study supervisor in person (or through video-recording in the Netherlands), covering each child age band and certified assessors. Supervisors independently complete questionnaires being administered by the assessor and complete a fidelity checklist. Assessors are given feedback based on checklist score. Supervisors review quality assurance findings with the WHO biweekly, along with discussions with the subject matter experts for further resolution, as needed.

The GSED application software for data collection has built-in data range and consistency checks. Data managers review and resolve issues daily in consultation with the local field team and/or WHO team.

For peer review only - http://bmjopen.bmj.com/site/about/guidelines.xhtml

Sample size

Sample size determination was based on the primary aim of assessing the psychometric properties of the GSED. To have sufficient power to estimate measurement parameters (abilities and difficulties) needed to calculate the D-score and DAZ scores at baseline and to detect DIF of 1 logit with a power of $1-\beta = 0.90$ and a two-sided significance level of $\alpha = 0.05$, a sample of N=1248 per site is required. Given the rapidity of development of children at this age, the latent trait is longer than tends to be found in educational tests which focus on a narrower ability range. The easiest item in our tool "Does your child smile?" has a difficulty of -13.2 logits (1.1 on the D-score scale) and the most difficult item has a difficulty of 8.4 logits (88.86 on the D-Score scale), a 21.6 logit span. Thus, a one logit difference is not particularly large, given the length of the latent trait. This sample size was calculated via optimization of the sample size at i) each age/sex stratum and ii) overall on 1000 simulated datasets generated from parameters suggested by the Rasch GSED model. See Supplementary file S1 for additional details.

Statistical Analysis

To construct the scores for the GSED SF and LF, a Rasch model will be fitted and the item fit statistics (infit and outfit) will be assessed (26). Any items with unacceptable fit levels will be removed. Items will be screened for whether they exhibit unacceptable levels of measurement non-invariance (i.e., they have approximately equal difficulties) across countries and other contextual variables. Items exhibiting unacceptable DIF (using the logistic regression method) will be discarded sequentially, and the item response models will be refit using the remaining items. The expected a posteriori (EAP) method (27) will be applied to the final model to estimate the latent ability parameter (the D-score). Systematic deviations from unidimensionality will be tested by performing a principal components analysis on the residuals of the Rasch model. The method uses a

For peer review only - http://bmjopen.bmj.com/site/about/guidelines.xhtml

prior normal distribution with a mean set equal to the average proficiency at the child's age and a standard deviation of 5. The ability estimates will be used to estimate preliminary developmental percentile curves against age using a Generalized Additive Model for Location Scale and Shape (GAMLSS). Note that this application of EAP estimates underestimates the true variability in the population because EAP estimates – as any measurement – are always imprecise. In daily practice, analysts will compare other EAP estimates to the reference. To support this type of application, we create the references from the EAP estimates and accept a (perhaps slight) underestimate of the true variability in child development in the population. Following previous methodology (28) software will be written to calculate DAZ-scores based on the final dataset in R (29), and a user-friendly front-end version created in R (ShinyApp) (30) and/or Excel.

Reliability (inter-rater and test-retest) for all GSED measures will be analyzed using ICC (at the score level) and Gwet's AC1 agreement (at the item level) statistics with 95% confidence intervals to determine whether items perform reliably within and between assessors (31). A cut-off value of 0.4 and above will be used to flag items as adequately reliable. Those items with agreement between 0.4 and 0.5 will be discussed to determine if modifications can be made to improve their administration and/or comprehension.

DAZ scores from the GSED SF and LF will be used to conduct validity analyses to ensure that the measures are capturing the construct they are purported to measure (construct validity). Concurrent validity will be assessed by correlating age-corrected Bayley-III or Griffiths Mental Development Scales scores with GSED DAZ scores. We anticipate that these scores will have low to moderate positive correlations. Convergent validity will be supported by statistically significant positive correlations (with 95% confidence interval) between the GSED scores and continuous contextual measures with prior evidence of association with child development. Comparisons between "known

For peer review only

groups" will be made using the following variables: maternal education, home learning opportunities, home environment, socioeconomic status (SES), maternal mental health and child anthropometry, and stunting to determine if scores discriminate between high and low categories for each variable using mean DAZ scores.

GSED scores at baseline and follow up will be correlated for predictive validity (positive association between baseline and at 6 months) and mixed-effects linear regression used to adjust for other contextual covariates and baseline scores.

Secondary (Exploratory) Aims

Reference Scores

We plan to develop a set of preliminary reference scores to facilitate comparison of DAZ scores across countries. From the full validation study sample, a sub-sample of children who have not experienced prior exposure to major known biological and environmental risk factors is selected (i.e., "reference sub-sample") (Table 3). Such an approach relies on the assumption that the attainment of basic developmental milestones captured by the GSED of children who are free of major risk factors is relatively similar globally (32). To develop the reference scores, we will fit GAMLSS (33) to flexibly model both conditional means, conditional standard deviations of scores, and, if necessary, conditional skewness and kurtosis. We will test our assumption that the distribution of scores is equivalent across sites by adding a site indicator at each moment of the distribution, and testing site effects for their statistical significance. Where possible, we will conduct standardization of scores to assist with the interpretation of scores by pooling data across countries. We will report the corresponding parameters of the GAMLSS model at appropriate ages.

Table 3. "Reference" sub-sample exclusion criteria (healthy sub-sample)

For peer review only - http://bmjopen.bmj.com/site/about/guidelines.xhtml

Sample	Exclusion criteria
Minimum sub-sample of “reference” children per site<br>N=522	1. Below secondary maternal education (<6 years of schooling)<br>2. Birthweight less than 2500 gr<br>3. Gestational age < 37 completed weeks (259 days) and ≥ 42 completed weeks (294 days) [assessed by ultrasound]<br>4. Undernutrition (weight for age, length for age, OR weight for height Z score of less than –2 on the WHO Child Growth Standards) at the time of developmental assessment<br>5. Known severe congenital birth defect<br>6. History of birth asphyxia OR neonatal sepsis requiring hospitalization<br>7. Known neurodevelopmental disorder/ disability (Severe visual problems, seizures, hearing impairment) OR other chronic health problems (that is congenital heart disease)

Adaptive testing

We will determine whether adaptive testing is a feasible and valid option to measure child development within the GSED (Box 2). Adaptive testing (34) is an administration method that continually adapts to the level of the child’s performance, thereby reducing test administration time. Previous simulations (35) indicated that theoretically substantial gains in the precision of scores are possible when using adaptive testing even if administering fewer items.

**Box 2: Adaptive testing validation methodology**
We investigate the feasibility by applying adaptive testing in addition to the traditional “fixed” GSED administration methods in the sub-sample designated for predictive validity analyses (N=502 per site) in three sites. The adaptive test is executed using tablets that are specially programmed to continually adjust child’s score after each item is administered, and to suggest the next item based on the answers already received (e.g., a more difficult item for a child with a higher score, an easier item for a child

For peer review only

For peer review only - http://bmjopen.bmj.com/site/about/guidelines.xhtml

with a lower score). Once the program establishes a reliable score, the administration is terminated. Both the adaptive test and the fixed test are administered with the same sub-sample during two separate visits alternating the order of administration to investigate the difference between the two modes of administration. We will investigate the following: the variance of user experience as a function of the average difficulty of milestones (leniency); the comparison of the D-score distribution under the adaptive testing procedure with the D-score distribution under the fixed GSED administration (using a z-test to assess the equivalence of the two modalities and plotting the results to show the level of concordance); and relation of the difference between the two D-scores to background variables.

Psychosocial Form

The PF measure is in an early stage and will undergo exploratory and confirmatory factor analyses to assess the internal scale structure. Associations between items and factor scores with variables suggesting a high risk of psychosocial stress, such as family resilience, social support, and family and community violence, in addition to GSED SF and LF scores (concurrent validity measures) will be examined.

ETHICS AND DISSEMINATION

The study complies with the International Ethical Guidelines for Biomedical Research Involving Human Subjects (36) and received ethical approval from the appropriate body in each site [Bangladesh – Projahnmo Research Foundation Institutional Review Board; Brazil – University Hospital, São Paulo (HU-USP); Cote d'Ivoire – Comite National D'Ethique des Sciences de la Vie et de la Sante (CNESVS); Pakistan – The Aga Khan University Ethics Review Committee; The Netherlands – Institutional Review Board TNO, Netherlands Organisation for Applied Scientific Research; The Republic of China

For peer review only - http://bmjopen.bmj.com/site/about/guidelines.xhtml

– IRB of Shanghai Children's Medical Center Affiliated to Shangai Jiao Tong University School of Medicine; United Republic of Tanzania – Zanzibar Health Research Institute] and within WHO (protocol GSED validation 004583 approved on 20.04.2020). The findings of the study will be disseminated following a comprehensive dissemination strategy to reach a diverse range of stakeholders at the local, national and international level.

DISCUSSION

For peer review only

The validation of the GSED SF and LF is a meticulous and systematic global process that introduces an innovative common metric (the D-score) that countries can use to track the progress of child development among populations of young children and will enable countries to adapt, modify, and evaluate their policies and programs to ensure that young children are effectively and equitably reaching their development potential and building the human capital needed for sustainable development. Additional attention is required on understanding young children's responses to psychosocial challenges within global contexts. The exploration of the GSED PF introduces an important opportunity to capture the non-normative developmental patterns among young children that are potential precursors to behaviour and psychiatric problems. The GSED validation has several important design, methodological and implementation characteristics that illustrate the rigour required to validate instruments to measure child development globally. First, it is conducted in seven countries with multiple linguistic, cultural and socioeconomic backgrounds. Second, GSED is implemented through an app-based data collection system that facilitates the implementation by reducing recording and transcribing errors and other common pitfalls of paper-based instruments. Third, this study builds on the best practices in validation by including a broad spectrum of psychometric methodologies (concurrent, predictive, convergent, and discriminant validity, test-retest and inter-rater

For peer review only - http://bmjopen.bmj.com/site/about/guidelines.xhtml

reliability, differential item functioning, and differential test functioning). Fourth, a secondary aim builds the evidence for the creation of preliminary reference scores for the SF and LF, based on a sub-sample with minimal exposure to major biological risk factors and to the extent possible, minimal social and environmental risk factors. Fifth, we are validating an adaptive testing design that can streamline administration by tailoring and reducing the number of items required to obtain a valid score. Sixth, we are testing a new measure of young children's non-normative psychosocial development.

One notable difference between the GSED SF and LF measures and other instruments of early child development is that the GSED measures are based on a unidimensional model of development through measurement approaches that are universally applicable across cultures. The measures do not follow the common multidimensional approach with separate scores for different domains or contexts. Our validation study intends to demonstrate that this model provides valid, reliable, and interpretable data globally. The GSED SF and LF may exclude some items that measure development in cultural or setting-specific ways, because the focus is on selecting items that are meaningful for understanding child development within any given setting. If specific aspects need to be captured locally, to increase cultural relevance we suggest that the GSED measures are lightly adapted with country or culture-specific item props (in agreement with WHO) and/or through the administration of additional measures.

There are several limitations to our study. Although we are validating the GSED in seven countries, including one high income setting, three sites are resource-limited (Bangladesh, Pakistan and United Republic of Tanzania). Additional evidence may be needed in high income countries to expand the validity and reliability of the GSED to population-representative samples in additional countries. Second, the GSED has been created using items that fit a Rasch model demonstrating developmental progress across

For peer review only

For peer review only - http://bmjopen.bmj.com/site/about/guidelines.xhtml

ages 0-3 years (9). This univariate model makes strict assumptions and may exclude items that do not show strong age gradients or items that measure development in a culturally-specific ways. Third, GSED was developed to address population and programmatic level evaluations of early child development globally. The GSED is presently not being validated for screening or diagnosing individual children. Finally, our three secondary aims are exploratory, and will require further research, including developing global standards to replace our preliminary reference scores with more specific global norms, as in the Multi-country Growth Reference Standards for children's weight and height. In the future we plan to collect additional data from countries using strict inclusion/exclusion criteria (e.g., additional considerations around environmental risk and protective factors) to further validate our initial reference scores. Similarly, we plan to conduct further work to explore the functionality, reliability, validity, and invariance of the PF. Lastly, as the GSED SF and LF scores are meant to be interpreted and used for population-level measurement, we plan to expand the work towards understanding of how the GSED package could be modified and validated to be able to identify individual children at risk of developmental delays and disorders.

For peer review only

LIST OF ABBREVIATIONS

Bayley-III	Bayley Scales of Infant and Toddler Development
BRS	Brief Resilience Scale
CI	Confidence interval
CPAS	Childhood Psychosocial Adversity Scale
DAZ	Development for Age Z-score
DHS	Demographic and Health Surveys
DIF	Differential item functioning
DTF	Differential test functioning
D-score	Developmental Score
EAP	Expected a posteriori
ECD	Early child development
ECDI 2030	Early Childhood Development Index 2030
FCI	Family Care Indicators
FSS	Family Support Scale
GAMLSS	Generalized Additive Model for Location Scale and Shape
GSED	Global Scales for Early Development
HAZ	Height-for-age z-score
HOME	Home Observation for Measurement of the Environment Inventory
ICC	Intraclass correlation coefficient
LF	Long Form
LMIC	Low- and middle-income country
MICS	Multiple Indicator Cluster Surveys
OSF	Open Science Framework
PF	Psychosocial Form
PHQ-9	The Patient Health Questionnaire-9
SDG	Sustainable Development Goals
SES	Socioeconomic status
SF	Short Form
SOPs	Standard operating procedures
WAZ	Weight-for-age z-score

For peer review only

582 **STATEMENTS**

583 **Ethics approval**: The study complies with the International Ethical Guidelines for
584 Biomedical Research Involving Human Subjects and received ethical approval from the
585 appropriate Ethics Review Committee (ERC) at the World Health Organization (WHO)
586 (protocol ID GSED validation 004583 approved on 20.04.2020) and in each site
587 [Bangladesh – Projahnmo Research Foundation Institutional Review Board; Brazil –
588 University Hospital, São Paulo (HU-USP); Cote d'Ivoire – Comite National D'Ethique
589 des Sciences de la Vie et de la Sante (CNESVS); Pakistan – The Aga Khan University
590 Ethics Review Committee; The Netherlands – Institutional Review Board TNO,
591 Netherlands Organisation for Applied Scientific Research; The Republic of China – IRB
592 of Shanghai Children's Medical Center Affiliated to Shangai Jiao Tong University
593 School of Medicine; United Republic of Tanzania – Zanzibar Health Research Institute].

594 **Patient consent:** Written consent is gathered by all study participants. Informed consent
595 forms are written to be easily understood by lay persons, enabling them to understand the
596 aims, procedures and potential risks of participation and have been approved by the
597 WHO ERC. For participants who are illiterate, culturally acceptable options including
598 witnessed oral consent and a thumbprint in lieu of a signature are accepted by the WHO
599 and local ERCs.

600 **Data sharing**: Not applicable (protocol paper)

601 **Author Contributions:** All authors contributed substantively to this work. VC was the
602 lead author in drafting the manuscript in addition to the technical contributions to the
603 study protocol conceptualization and development. TD led the conceptualization of the
604 study, MG, MMB, MJ, and PK contributed significantly to the conceptualization of the
605 study design and methodology, drafted sections of the protocol and related manuscript;
606 GL, GMc, [focus on psychometric properties], DMc, JS [focus on preliminary reference

For peer review only

scores] MW [focus on testing of psychosocial form], SvB and IE [focus on adaptive testing] came to consensus on statistical analysis plan, determined the sample size calculations, and drafted the related parts of the manuscript relevant to their specific expertise ; AN, AR, KH, and AW drafted substantial pieces of the manuscript related to sampling frame, study measures and implementation. SA, AD, RA, AB, FJ, YS, IN, RK, SS, AZ, MPM, YZ, FT, ARD, AB, JZ, AH, GF, SD, NSK, FB, FJ, and MRC contributed to the adaptation of the study protocol for feasibility and on-the-ground implementation, focusing on manuscript write up related to site-specific descriptions. All above authors, in addition to RK, MMP, and RN reviewed and edited the study protocol and the manuscript. All authors read and approved the final manuscript submission.

Acknowledgements: None

For peer review only - http://bmjopen.bmj.com/site/about/guidelines.xhtml

COMPETING INTERESTS

The authors declare that they have no competing interests.

FUNDING

This work was supported (alphabetical order) by Bernard van Leer Foundation, Bill & Melinda Gates Foundation, Children's Investment Fund Foundation, Jacobs Foundation and King Baudouin Foundation, United States. The funders provided financial support. The design, implementation, and writing of the manuscript were led by the World Health Organization.

For peer review only

For peer review only - http://bmjopen.bmj.com/site/about/guidelines.xhtml

REFERENCES:

1. Clark H, Coll-Seck AM, Banerjee A, Peterson S, Dalglish SL, Ameratunga S, et al. A Future for The World's Children? A WHO–UNICEF–Lancet Commission. The Lancet. 2020;395(10224):605-58.
2. UnitedNations. https://sdgs.un.org/goals/goal4 [cited 2021 November]. Department of Economic and Social Affairs, Sustainable Development].
3. Fernald LC, Prado E, Kariger P, Raikes A. A Toolkit for Measuring Early Childhood Development in Low- and Middle-Income Countries. Washington DC 20433: International Bank for Reconstruction and Development / The World Bank; 2017.
4. UNICEF. https://data.unicef.org/resources/early-childhood-development-index-2030-ecdi2030/ [updated January 2021].
5. Faruk T, King C, Muhit M, Islam MK, Jahan I, Baset KU, et al. Screening tools for early identification of children with developmental delay in low- and middle-income countries: a systematic review. BMJ Open. 2020;10(11):e038182.
6. Weber AM, Rubio-Codina M, Walker SP, Van Buuren S, Eekhout I, Grantham-McGregor SM, et al. The D-Score: A Metric for Interpreting The Early Development of Infants and Toddlers across Global Settings. BMJ Global Health. 2019;4(6):e001724.
7. McCoy DC, Waldman M, Team CF, Fink G. Measuring Early Childhood Development at a Global Scale: Evidence from the Caregiver-Reported Early Development Instruments. Early Childhood Research Quarterly. 2018;45:58-68.
8. GSEDTeam. A New Measure of Development in Children from Birth to Age 3 at Population Level: The Global Scale for Early Development (GSED). Early Childhood Matters. 2019.
9. Van Buuren S, Eekhout I, McCray G, Waldman M, Lancaster G, Black M, et al. A Scale for Tracking Child Development from Directly Observed and Caregiver-Reported Data. [Manuscript in preparation]
10. McCray G, McCoy D, Kariger P, Janus M, Black M, Chang-Lopez S, et al. The Creation of the Global Scales of Early Development (GSED) for 0-3 year old Children: Combining Subject Matter Expert judgements with big data. [Manuscript in preparation]
11. Van Buuren S, Eekhout I. Child development with the D-score: turning milestones into measurement [version 1; peer review: 1 approved with reservations]. Gates Open Research. 2021;5(81):1-75.
12. Rasch G. Probabilistic Models for Some Intelligence and Attainment Tests. MESA Press, 5835 S. Kimbark Ave., Chicago: ERIC; 1993.
13. Nizar A, Kaur R, Gladstone M, Lancaster G, Baqui A, Cavallera V, et al. Assessment of Acceptability and Feasibility of the Global Scales for Early Development (GSED) Across Three Settings for 0 to 3-year-old Children. [Manuscript in preparation]
14. Streiner DL, Norman GR, Cairney J. Health measurement scales: a practical guide to their development and use: Oxford University Press, USA; 2015.
15. Lancaster GA, Kariger P, McCray G, Janus M, Gladstone M, Cavallera V, et al. Conducting a Feasibility Study in a Global Health Setting for Constructing a Caregiver-Reported Measurement Tool: An Example in Infant and Young Child Development: SAGE Publications Ltd; 2020.
16. McCoy DC, Sudfeld CR, Bellinger DC, Muhihi A, Ashery G, Weary TE, et al. Development and Validation of an Early Childhood Development Scale for Use in Low-Resourced Settings. Population Health Metrics. 2017;15(1):1-18.
17. Walker SP, Wachs TD, Gardner JM, Lozoff B, Wasserman GA, Pollitt E, et al. Child Development: Risk Factors for Adverse Outcomes in Developing Countries. The Lancet. 2007;369(9556):145-57.

For peer review only

For peer review only - http://bmjopen.bmj.com/site/about/guidelines.xhtml

18. Jones PC, Pendergast LL, Schaefer BA, Rasheed M, Svensen E, Scharf R, et al. Measuring Home Environments Across Cultures: Invariance of the HOME Scale Across Eight International Sites from the MAL-ED Study. Journal of School Psychology. 2017;64:109-27.

19. Kariger P, Frongillo EA, Engle P, Britto PMR, Sywulka SM, Menon P. Indicators of Family Care for Development for Use in Multicountry Surveys. Journal of Health, Population, and Nutrition. 2012;30(4):472.

20. Berens AE, Kumar S, Tofail F, Jensen SK, Alam M, Haque R, et al. Cumulative Psychosocial Risk and Early Child Development: Validation and Use of the Childhood Psychosocial Adversity Scale in Global Health Research. Pediatric Research. 2019;86(6):766-75.

21. Smith BW, Dalen J, Wiggins K, Tooley E, Christopher P, Bernard J. The Brief Resilience Scale: Assessing the Ability to Bounce Back. International Journal of Behavioral Medicine. 2008;15(3):194-200.

22. Dunst C. The Family Support Scale: Reliability and Validity. Journal of Individual, Family, and Community Wellness. 1984;1(4):45-52.

23. Moriarty AS, Gilbody S, McMillan D, Manea L. Screening and Case Finding for Major Depressive Disorder Using the Patient Health Questionnaire (PHQ-9): A Meta-Analysis. General Hospital Psychiatry. 2015;37(6):567-76.

24. Bayley N. Bayley Scales of Infant and Toddler Development: Administration Manual,. 3rd edition ed. United States of America: Psychorp; 2006.

25. Jacklin L, Cockcroft K. The Griffiths Mental Developmental Scales: An Overview and a Consideration of their Relevance for South Africa. In: Laher S, Cockcroft K, editors. Psychological Assessment in South Africa. Research and applications: Wits University Press; 2013. p. 169-85.

26. Wright BD, Masters GN. Rating scale analysis: Rasch Measurement. Chicago, IL.: MESA press; 1982.

27. Bock RD, Mislevy RJ. Adaptive EAP Estimation of Ability in a Microcomputer Environment. Applied Psychological Measurement. 1982;6(4):431-44.

28. Gladstone M, Lancaster G, McCray G, Cavallera V, Alves CR, Maliwichi L, et al. Validation of the Infant and Young Child Development (IYCD) Indicators in Three Countries: Brazil, Malawi and Pakistan. International Journal of Environmental Research and Public Health. 2021;18(11):6117.

29. Team RC. R: A language and environment for statistical computing. R Foundation for Statistical Computing, Vienna, Austria. http://wwwR-projectorg/. 2016.

30. Winston Chang, Joe Cheng, JJ Allaire, Carson Sievert, Barret Schloerke, Yihui Xie, Jeff Allen, Jonathan McPherson, Alan Dipert, Barbara Borges. shiny: Web Application Framework for R. R package version 1.7.2 2022 [Available from: https://CRAN.R-project.org/package=shiny. .

31. Gwet KL. Computing Inter-Rater Reliability and its Variance in the Presence of High Agreement. British Journal of Mathematical and Statistical Psychology. 2008;61(1):29-48.

32. Ertem IO, Krishnamurthy V, Mulaudzi MC, Sguassero Y, Balta H, Gulumser O, et al. Similarities and Differences in Child Development from Birth to Age 3 years by Sex and Across Four Countries: A Cross-sectional, Observational Study. The Lancet Global Health. 2018;6(3):e279-e91.

33. Rigby RA, Stasinopoulos DM. Generalized Additive Models for Location, Scale and Shape. Journal of the Royal Statistical Society: Series C (Applied Statistics). 2005;54(3):507-54.

34. Wainer H, Dorans NJ, Flaugher R, Green BF, Mislevy RJ. Computerized Adaptive Testing: A Primer: Routledge; 2000.

35. Jacobusse G, Van Buuren S. Computerized Adaptive Testing for Measuring Development of Young Children. Statistics in Medicine. 2007;26(13):2629-38.

36. Singh AN. Current Status of Research Ethics and Projecting Future Initiatives. International Medical Journal. 2018;25(6).

For peer review only - http://bmjopen.bmj.com/site/about/guidelines.xhtml

For peer review only

For peer review only - http://bmjopen.bmj.com/site/about/guidelines.xhtml

726 **FIGURE LEGEND**

727 **Figure 1. Development chart**

728 Reproduced with permission from van Buuren S and Eekhout I (2021) (11)

729

730 **Figure 2. Study Sampling schema diagram**

For peer review only

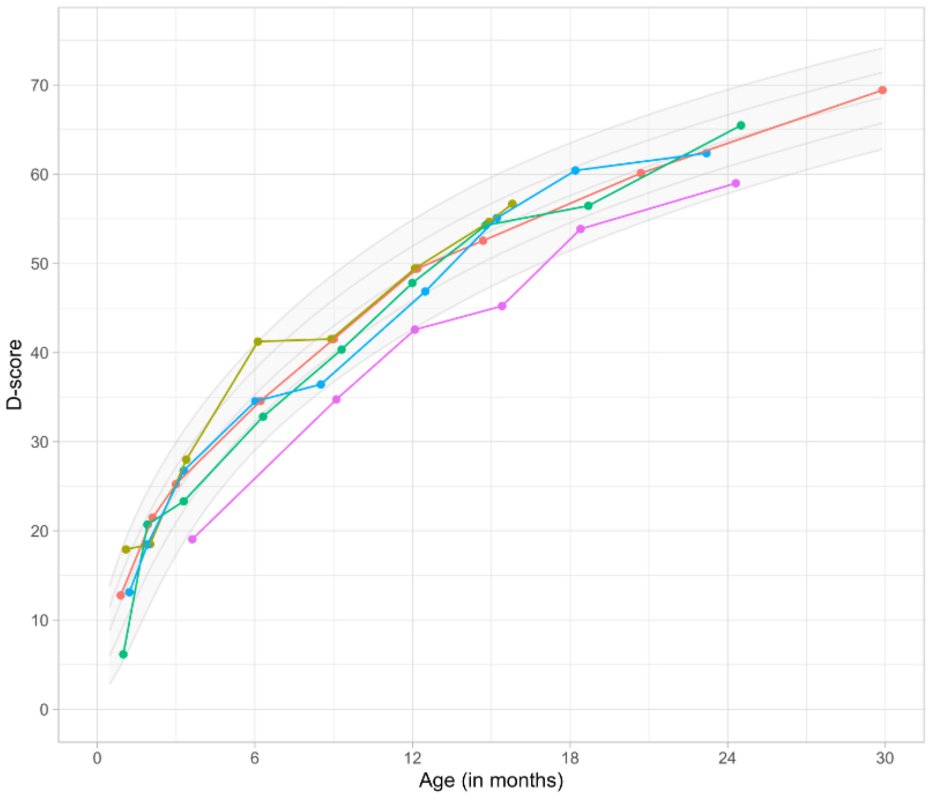

152x125mm (144 x 144 DPI)

GSED study sample

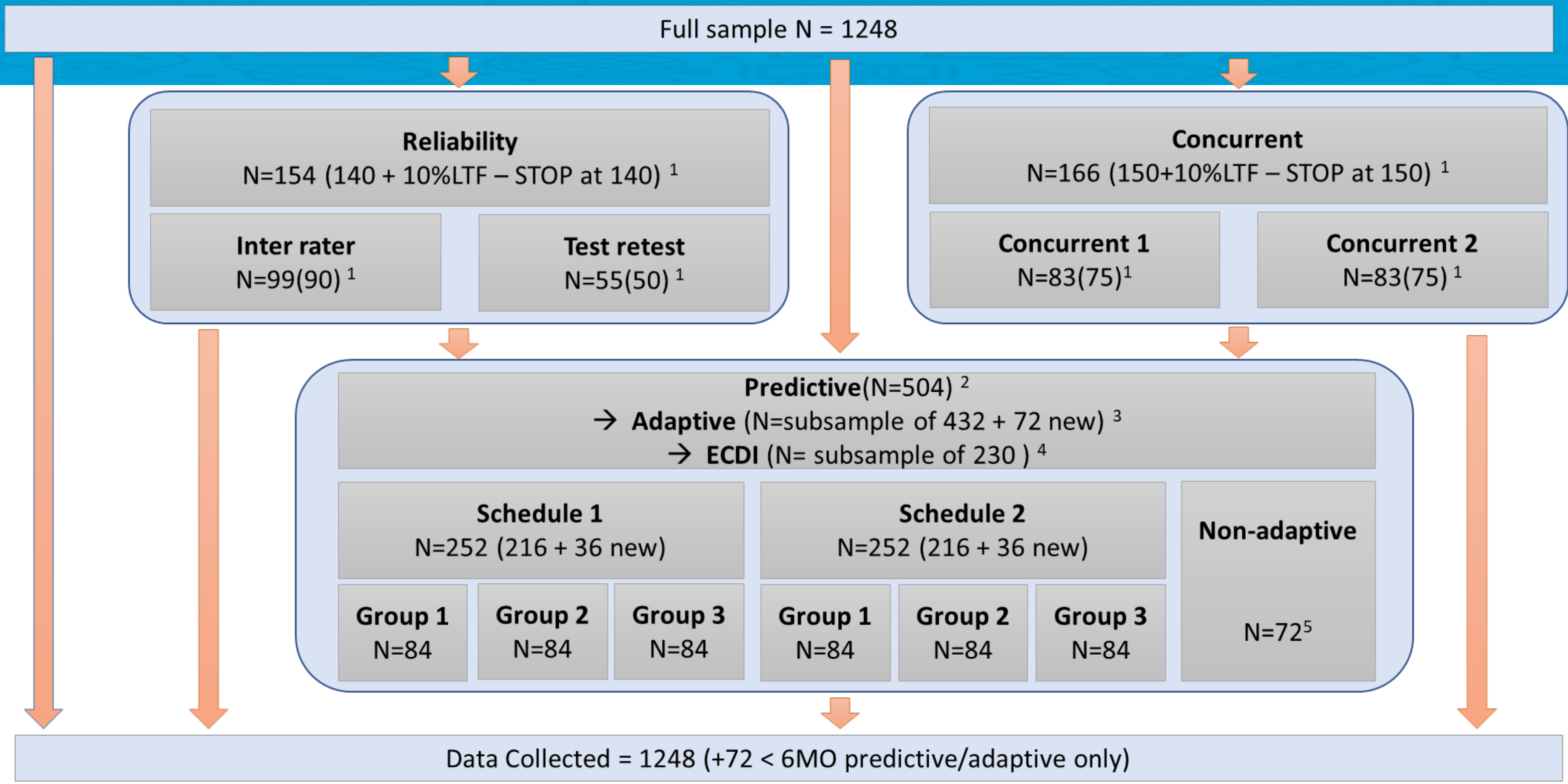

[1] The number inside parentheses is the number collected and the number outside is the number randomised to account for loss to follow-up

[2] Two additional participants have been added to the predictive to have equal numbers in each experimental group.

[3] 72 new children between 2 weeks and 6 months of age have been added to the adaptive sample to ensure coverage at the lower ages.

[4] ECDI will only be done on N=230 Children between the ages of 2+ years at the time of the predictive data collection.

[5] The 72 oldest children (36-41 months) from the predictive sample will not be part of the adaptive sample.

For peer review only - http://bmjopen.bmj.com/site/about/guidelines.xhtml

Supplementary file 1 – Sample size calculations and sampling frame

The sample size calculation for reliability is based on a confidence interval (CI) approach and the desired accuracy for the lower bound of the CI for the ICC estimates. In an Analysis of Variance (ANOVA) with 2-way random effects on a single score with 2 observations per subject (following Shrout and Fleiss, 1979) (27) and with a two-sided 95% confidence interval and an expected ICC of 0.875, the lower confidence interval for the inter-rater reliability sample (N=90) = 0.852. With the same parameters but an expected ICC of 0.90 the lower confidence interval for the test-retest reliability sample (N=50) is 0.830. We expect the ICC to be higher for the test-retest reliability than the inter-rater reliability as inter-rater estimates contain all the sources of error in the test-retest estimates, plus additional error between assessors (14).

To assess concurrent validity, a sample size of 150 per site produces a two-sided 95% CI 0.15-0.44, when the estimate of Pearson's product-moment correlation is 0.30, with an equal spread of participants tested across age and sex. The CI will be narrower when the data are combined across all seven countries. To assess predictive validity a sample size of 404 produces a two-sided 95% CI 0.65-0.75 when the estimate of Pearson's product-moment correlation is 0.70 between individual scores at baseline and at the 6-month follow-up. Allowing 20% dropout at follow up, a sample size of approximately 500 participants is required.

Table S1. Sampling Frame

Sample size per site by age and sex for total population (n=1248) which includes a minimum subsample of healthy 'reference' children (n=522)

Age (Days)	Sex	Total Sample size	Minimum sub-sample of reference children	Predictive validity sample (6-month follow-up; age at baseline)	Reliability: Inter-rater	Reliability: Test-Retest	Concurrent validity
15-30	Male	40	20	8	2	1	4
	Female	40	20	8	2	1	2
31-61	Male	40	12	8	1	1	2
	Female	40	12	8	2	1	2
62-91	Male	40	10	8	2	1	2
	Female	40	10	8	1	0	4
92-122	Male	36	9	8	2	1	2
	Female	36	9	8	2	1	2
123-152	Male	32	8	8	1	1	2
	Female	32	8	8	2	1	2

For peer review only

153-183	Male	28	8	8	1	0	4
	Female	28	8	8	1	1	2
184-213	Male	25	7	8	2	1	2
	Female	25	7	8	1	0	2
214-244	Male	23	7	8	1	1	2
	Female	23	7	8	2	1	4
245-274	Male	21	6	8	1	1	2
	Female	21	6	8	1	1	2
275-304	Male	19	6	8	2	0	2
	Female	19	6	8	1	1	2
305-335	Male	17	6	8	1	1	4
	Female	17	6	8	2	0	2
336-365	Male	16	6	7	1	1	2
	Female	16	6	7	1	1	2
366-396	Male	14	6	7	2	1	2
	Female	14	6	7	1	1	4
397-426	Male	13	6	7	1	0	2
	Female	13	6	7	2	1	2
427-457	Male	12	5	7	1	1	2
	Female	12	5	7	1	0	2
458-487	Male	11	5	7	2	1	4
	Female	11	5	7	1	1	2
488-517	Male	11	5	7	1	1	2
	Female	11	5	7	2	1	2
518-548	Male	10	5	7	1	0	2
	Female	10	5	7	1	1	4
549-578	Male	9	5	7	2	1	2
	Female	9	5	7	1	0	2
579-609	Male	9	5	7	1	1	2
	Female	9	5	7	2	1	2
610-639	Male	9	5	7	1	1	4
	Female	9	5	7	1	1	2
640-670	Male	9	5	7	2	0	2
	Female	9	5	7	1	1	2
671-700	Male	9	5	7	1	1	2
	Female	9	5	7	2	0	4
701-730	Male	9	5	7	1	1	2
	Female	9	5	7	1	1	2
731-761	Male	9	5	7	2	1	2
	Female	9	5	7	1	1	2
762-791	Male	9	5	6	1	0	4
	Female	9	5	6	2	1	2
792-822	Male	9	5	6	1	1	2
	Female	9	5	6	1	0	2
823-852	Male	9	5	6	2	1	2
	Female	9	5	6	1	1	2

853-883	Male	9	5	6	1	1	2
	Female	9	5	6	2	1	2
884-913	Male	9	5	6	1	0	2
	Female	9	5	6	1	1	2
914-944	Male	9	5	6	2	1	2
	Female	9	5	6	1	0	2
945-974	Male	9	5	6	1	1	2
	Female	9	5	6	2	1	2
975-1004	Male	9	5	6	1	1	2
	Female	9	5	6	1	1	2
1005-1035	Male	9	5	6	2	0	2
	Female	9	5	6	1	1	2
1036-1065	Male	9	5	6	1	1	2
	Female	9	5	6	2	0	2
1066-1096	Male	9	5	6	1	1	2
	Female	9	5	6	1	1	2
1097-1126	Male	9	5	0	0	0	0
	Female	9	5	0	0	0	0
1127-1157	Male	9	5	0	0	0	0
	Female	9	5	0	0	0	0
1158-1187	Male	9	5	0	0	0	0
	Female	9	5	0	0	0	0
1188-1218	Male	9	6	0	0	0	0
	Female	9	6	0	0	0	0
1219-1248	Male	9	6	0	0	0	0
	Female	9	6	0	0	0	0
1249-1279	Male	9	7	0	0	0	0
	Female	9	7	0	0	0	0
**TOTAL**		1248	522	504	*99	**55	***166

*90 + ~10% Loss to follow up = 99; **50 + ~10% Loss to follow up = 55; ***150 + ~10% Loss to follow up = 166

For peer review only

Supplementary file 2 – Visit schedule

Table S2a.Visit Schedule for the GSED Validation Study (all sites except the Netherlands)

**Main Study Only [No Sub-sample]**	**Inter- Rater Reliability Sub- Sample**	**Test- Retest Reliability Sub- Sample**	**Concurrent Sub- Sample 1 [LF First]**	**Concurrent Sub- Sample 2 [BSID III First]**
**Visit 1 [At Home]**				
Eligibility and Consent	Eligibility and Consent	Eligibility and Consent	Eligibility and Consent	Eligibility and Consent
COVID Questionnaire	COVID Questionnaire	COVID Questionnaire	COVID Questionnaire	COVID Questionnaire
Contextual	Contextual	Contextual	Contextual	Contextual
GSED Short form [SF]	GSED Short form [SF]	GSED Short form [SF]	GSED Short form [SF]	GSED Short form [SF]
GSED Psychosocial form [PF]	GSED Psychosocial form [PF]	GSED Psychosocial form [PF]	GSED Psychosocial form [PF]	GSED Psychosocial form [PF]
HOME Inventory or Family Care Indicators (FCI)	HOME Inventory or Family Care Indicators (FCI)	HOME Inventory or Family Care Indicators (FCI)	HOME Inventory or Family Care Indicators (FCI)	HOME Inventory or Family Care Indicators (FCI)
Anthropometrics*	Anthropometrics*	Anthropometrics*	Anthropometrics*	Anthropometrics*
**Visit 2 [At home, clinic, or other setting within 48 hours of visit 1]** ***Note: For Concurrent Sample, the Visit is at the Clinic setting***				
Abbreviated Eligibility [Coversheet]	Abbreviated Eligibility [Coversheet]	Abbreviated Eligibility [Coversheet]	Abbreviated Eligibility [Coversheet]	Abbreviated Eligibility [Coversheet]
GSED Long form [LF]	GSED Long form [LF]	GSED Long form [LF]	**GSED Long form [LF]**	**BSID III**
CPAS	CPAS	CPAS	CPAS	----------------------
PHQ9	PHQ9	PHQ9	PHQ9	---------------------
Family support & Resilience Scale	Family support & Resilience Scale	Family support & Resilience Scale	Family support & Resilience Scale	----------------------
**Visit 3 [Setting and timing vary by sub-sample]**				
Visit 3 not required	Visit 3 [At home, clinic or other setting where the LF was completed- **within 24** hours of the LF]	Visit 3 [At home, clinic or other setting where the LF was completed- this should happen **7 to 10 days after** LF]	Visit 3 [Clinic setting within **24- 72 hours** of the LF- can be done at same time as Visit 2 – taking child fatigue into consideration]	Visit 3 [Clinic setting within **24- 72 hours** of the BSID III - can be done at same time as Visit 2 – taking child fatigue into consideration]
	Abbreviated Eligibility [Coversheet]	Abbreviated Eligibility [Coversheet]	Abbreviated Eligibility [Coversheet]	Abbreviated Eligibility [Coversheet]
	GSED Short form [SF]	GSED Short form [SF]	**BSID III**	**GSED Long form [LF]**
	GSED Psychosocial form [PF]	GSED Psychosocial form [PF]	----------------------	CPAS
	GSED Long form [LF]	GSED Long form [LF]	----------------------	PHQ9
	----------------------	----------------------	----------------------	Family support & Resilience Scale

* Anthropometrics may be done either at visit 1 or visit 2

Table S2b: Visit Schedule for the GSED Validation Study (the Netherlands only)

**Main Study Only [No Sub-sample]**	**Inter- Rater Reliability Sub- Sample**	**Test- Retest Reliability Sub- Sample**	**Concurrent Sub- Sample 1 [LF First]**	**Concurrent Sub- Sample 2 [BSID III First]**
**Session 1 [Online]**				
Eligibility and Consent	Eligibility and Consent	Eligibility and Consent	Eligibility and Consent	Eligibility and Consent
Contextual	Contextual	Contextual	Contextual	Contextual
GSED Short form [SF]	GSED Short form [SF]	GSED Short form [SF]	GSED Short form [SF]	GSED Short form [SF]
GSED Psychosocial scale [PS]	GSED Psychosocial scale [PS]	GSED Psychosocial scale [PS]	GSED Psychosocial scale [PS]	GSED Psychosocial scale [PS]
**Visit 1 [At clinic within 48 hours of session1]**				
Abbreviated Eligibility [Coversheet]	Abbreviated Eligibility [Coversheet]	Abbreviated Eligibility [Coversheet]	Abbreviated Eligibility [Coversheet]	Abbreviated Eligibility [Coversheet]
GSED Long form [LF]	GSED Long form [LF]	GSED Long form [LF]	**GSED Long form [LF]**	**BSID III**
Anthropometrics	Anthropometrics	Anthropometrics	Anthropometrics	Anthropometrics
**Session 2 [Online, Test-Retest of SF/PSY within 7 to 10 days of online session 1]**				
Abbreviated Eligibility [Coversheet]	Abbreviated Eligibility [Coversheet]	Abbreviated Eligibility [Coversheet]	Abbreviated Eligibility [Coversheet]	Abbreviated Eligibility [Coversheet]
COVID Questionnaire	COVID Questionnaire	COVID Questionnaire	COVID Questionnaire	COVID Questionnaire
----------------------	----------------------	GSED Short form [SF]	----------------------	----------------------
----------------------	----------------------	GSED Psychosocial scale [PS]	----------------------	----------------------
CPAS	CPAS	CPAS	CPAS	CPAS
PHQ9	PHQ9	PHQ9	PHQ9	PHQ9
Family support & Resilience Scale	Family support & Resilience Scale	Family support & Resilience Scale	Family support & Resilience Scale	Family support & Resilience Scale
Family Care Indicators (FCI)	Family Care Indicators (FCI)	Family Care Indicators (FCI)	Family Care Indicators (FCI)	Family Care Indicators (FCI)
**Visit 2 [At clinic, timing varies by sub-sample]**				
Visit 2 not required	Visit 2 [**within 24** hours of the LF]	Visit 2 [**7 to 10 days after** LF]	Visit 2 [within **24- 72 hours** of the LF- can be done at same time as Visit 1 – taking child fatigue into consideration]	Visit 2 [within **24- 72 hours** of the BSID III - can be done at same time as Visit 1 – taking child fatigue into consideration]
	Abbreviated Eligibility [Coversheet]	Abbreviated Eligibility [Coversheet]	Abbreviated Eligibility [Coversheet]	Abbreviated Eligibility [Coversheet]
	GSED Long form [LF]	GSED Long form [LF]	**BSID III**	**GSED Long form [LF]**
